# Decoding honey-sweet flavored flue-cured tobacco from Guizhou with data science and flavoromics by volatile and cell wall components

**DOI:** 10.3389/fchem.2025.1613828

**Published:** 2025-10-01

**Authors:** Risheng Zhong, Zhenchun Sun, Liang Feng, Haitao Chen, Shuqi Wang, Yechun Lin, Jie Sun, Ning Zhang, Huiying Zhang, Feng Wang

**Affiliations:** 1 Beijing Advanced Innovation Center for Food Nutrition and Human Health, Beijing Key Laboratory of Flavor Chemistry, Beijing Technology and Business University (BTBU), Beijing, China; 2 Tobacco Science Institute of Guizhou Province, Guiyang, Guizhou, China

**Keywords:** flavor, volatile, flue-cured tobacco, aroma type, cell wall components, analysis

## Abstract

*Nicotiana tabacum* L. is often called tobacco. The aroma of flue-cured tobacco (FCT) varies according to the origin and grade. In this study, volatiles and plant cell wall components (CWC) were used to differentiate aroma types and grades of FCT, with a focus on the honey-sweet flavored FCT from Guizhou, China. Volatiles were analyzed by headspace solid-phase microextraction gas chromatography/mass spectrometry, while CWC (cellulose, hemicellulose, pectin, lignin) were quantified. Results indicated that upper leaf (Grade B) tobacco contained higher volatile levels than middle leaf (Grade C). Multivariate analyses-Principal component analysis (PCA), orthogonal partial least squares discriminant analysis (OPLS-DA), and logistic regression (LR) identified 27 key volatiles contributing to aroma differentiation in FCT origin. By combining the screened volatiles with odor activity value, the most important key aroma compounds that distinguish Guizhou honey-sweet flavored from other origins were β-cyclocitral and 1-nonanal. The CWC showed significant variation across origins or grades. Machine learning models (e.g., LR with 96.5% accuracy) effectively distinguished the origin of FCT. This study pioneers the integration of machine learning with molecular sensory science to decode the unique honey-sweet flavor of Guizhou flue-cured tobacco, addressing a critical gap in linking volatile biomarkers to regional terroir. This methodology provides a way to evaluate tobacco quality and aroma characteristics.

## 1 Introduction

*Nicotiana tabacum* L., commonly referred to as tobacco, is a common perennial plant belonging to the genus Nicotiana, family Solanaceae (Philip, 2014). Tobacco is one of the most widely grown cash crops in the world (Peedin, 2011). In addition to its use as a raw and processed material in the manufacture of cigarettes, tobacco has been employed as an insecticide, anesthetic, sedative, diuretic and analgesic (Hu Z. et al., 2021; Lei et al., 2013). China is the world’s largest producer of tobacco, in terms of both area cultivated and quantity produced (Martins-da-Silva et al., 2022). Tobacco leaves undergo a series of processes including roasting, leaf beating, re-roasting, and tobacco mellowing. The final product is a roasted tobacco with a unique aroma (Hu B. et al., 2021). The flavor of tobacco varies markedly among different production regions. In China, flue-cured tobacco (FCT) is categorized into eight distinct types based on their olfactory characteristics, with each type corresponding to a specific geographical area of production. The FCT in Guizhou is distinguished by its distinctive aroma type, which is characterized by a honey-sweet quality. The main characteristics are a prominent honey-sweet aroma, with the main notes being hay-like and honey-sweet. The auxiliary notes include a mellow-sweet, woody, fresh-sweet aroma, among others (Luo, 2019). Aroma can reveal the quality of the tobacco. Cell wall components (CWC) also significantly influence tobacco quality. The most prevalent CWC include cellulose, hemicellulose, pectin, and lignin (Zhao et al., 2019). A positive correlation has been demonstrated between CWC and the maturity, structure, identity, oil content and coloration of tobacco (Chen et al., 2011). It is evident that cellulose, pectin and lignin have a significant impact on the quality of tobacco (Liu, 2015). The CWC and volatiles present in tobacco have a significant impact on its usability and quality as a raw material for the subsequent processing of products. The detection of aroma and CWC collectively serves as an indicator of tobacco quality (Johannes and Wolf-Dieter, 2020).

The aroma of tobacco is typically derived from the degradation of terpenes, terpene alcohols, esters, lipids, alkaloids and other precursors, which are commonly found in phenylalanine degradation products, carotenoid degradation products and cedarane-like degradation products, amongst others (Fan et al., 2022; Geng et al., 2023; Liu A. et al., 2022). A range of methods exists for studying the volatiles of tobacco. Different pretreatment and detection methods are employed in different situations. In order to achieve optimal extraction outcomes, the process of solid-phase microextraction (SPME) was enhanced by employing Microwave-assisted deep eutectic-SPME and Microwave-assisted water eutectic-SPME, leading to the identification of 31 and 13 volatiles, respectively, which were not detected when SPME was utilized in its conventional form (Nie et al., 2017). A total of 81 volatiles were detected in Canadian and Yunnan-roasted tobacco samples. These were analyzed using conventional extraction methods in conjunction with distillation extraction (Tie et al., 2024). However, the vast majority of volatile substances are detected by headspace (HS)-SPME-GC/MS, which is the preferred method for volatile compounds and complex samples due to its solvent-free, cost-effective, convenient, rapid, relatively stable, multi-functional and high-throughput sample preparation characteristics (Jalili et al., 2020). For instance, a total of 58 volatiles were detected by SPME-GC-MS in tobacco from eight different regions (Wang et al., 2024), 56 volatiles were detected by SPME-GC-MS in 14 different types of tobacco from the Fujian production area (Gong et al., 2023), and a total of 40 volatiles were detected by SPME-GC-MS for volatiles during the fermentation of roasted tobacco leaves (Zhang et al., 2024).

Furthermore, molecular sensory science can be used to investigate the significance of various volatiles on aroma, frequently employing the odor activity value (OAV) to ascertain whether the concentration attains the olfactory threshold, thereby determining the compound’s effect on the aroma (Guadagni et al., 1966). This method is also used in tobacco aroma research (Zhao et al., 2025). In the field of data analysis, the integration of statistics and computing technology has enhanced the efficiency of data utilization, facilitating the identification of associations between variables such as geographical origin and sensory characteristics. The incorporation of chemometrics and machine learning algorithms into data processing has become a prevalent practice in the analysis of tobacco aroma. The evaluation model developed by Ye, X.F. et al. based on aroma quality and principal component analysis can more objectively, directly and accurately reflect the aroma quality of tobacco (Ye et al., 2011). Support Vector Machine (SVM) algorithm was used together to select 22 chemical compounds by Relief F-Particle Swarm Optimization to classify the tobacco leaves (Gu et al., 2016). Partial least squares regression (PLSR) analysis has the capacity to reveal that aroma precursors were positively correlated with the sensory quality characteristics of flue-cured tobacco (Li et al., 2024). These tools and methods have facilitated the identification of volatile compounds among the numerous volatiles that are more likely to have a significant impact on aroma. Despite extensive research on tobacco volatiles, the mechanistic link between region-specific aroma signatures and molecular drivers remains poorly understood.

The aim of this study is to identify the volatile compounds responsible for the honey-sweet flavor profile in FCT. Samples were collected from 11 production regions in Guizhou Province, China, and three neighboring provinces. A total of 28 samples were collected from each region, comprising upper leaves (Grade B) and middle leaves (Grade C). Volatile compounds were extracted from the tobacco leaves using HS-SPME/GC-MS technology and the CWC content was measured simultaneously. The study examined the discriminatory power of CWC content across different growing regions and grades. Chemometric methods and machine learning logistic regression were employed to investigate how the types and levels of volatile compounds distinguish origin and grade. The study integrated chemometrics, machine learning and molecular sensory science to identify the characteristic aroma compounds that distinguish Guizhou tobacco from that of other regions. The results suggest that the distinctive honey-like flavor profile of Guizhou tobacco is determined by specific volatile compounds.

## 2 Materials and methods

### 2.1 Materials preparation

The FCT were collected from 14 origins, with each origin contained upper leaf (Grade B) and middle leaf (Grade C), a total of 28 samples. The B and C grades are from different parts of the leaf, and all the color grades of the leaf are orange-yellow. All samples were provided by the Guizhou branch of China National Tobacco Corporation (CNTC). Samples were collected from representative farmers in key monitored townships, selecting households characterized by medium-to-high production levels, medium-sized operations, and stable tobacco cultivation. Three households were chosen from each monitoring area. The sampled variety was Yunyan 87, which was cut into fine shreds and uniformly blended. All samples can be categorized into two major origins, those within Guizhou province and those outside Guizhou province. Among the origins, HBNS, HNCZ and YNDL were tobacco leaves from outside Guizhou province, and the rest of the origins were samples from inside Guizhou province, as shown in Supplementary Tables SA1, SA2.

### 2.2 Chemicals

Normal alkanes C_6_∼C_30_ were purchased from Sigma-Aldrich Company, USA. deuterated hexanol-D13 was purchased from Beijing Quanpinsu Biotechnology Co., Ltd. Dichloromethane was purchased from Beijing InnoChem Science & Technology Co., Ltd. Helium was purchased from Beijing Beihe Pufen Gas Industry Co., Ltd. The reagents for CWC assay were purchased from Sinopharm Chemical Reagent Co.

### 2.3 Measurement of CWC

#### 2.3.1 Pectin measurement

The sample (0.1 g) was weighed and transferred into a centrifuge tube. The tube was then filled with steel beads and 1 mL of anhydrous ethanol. The contents of the tube were subsequently agitated using a grinding apparatus. The tube was placed into a heating block and subjected to a temperature of 90 °C for a duration of 10 min. The contents of the tube were subjected to a centrifugal force of 10,000 rpm for a duration of 3 min. Remove the upper layer of the mixture and leaving the precipitate. Repeat the following steps three times: add 70% ethanol at 80 °C, rinse the precipitate, centrifuge, remove the supernatant and leave the precipitate. Add 1 mL of sulfuric acid solution (pH = 0.5), heat at 90 °C for 1 h, and centrifuge at 10,000 rpm for 3 min. Aspirate 100 μL of supernatant and add 275 μL of concentrated sulfuric acid. Mix well and water bath at 90 °C for 15 min. Add 25 μL of carbazole ethanol solution and mix well. Then, react in the dark for 30 min. Aspirate 200 μL of supernatant on top of the enzyme plate, and then measure the absorbance value at 525 nm. Absorbance value was measured at 525 nm. At the same time, different concentrations of galacturonic acid solution was configured, and the standard curve was plotted (NY/T 2016-2011, 2025).

#### 2.3.2 Lignin measurement

A dried and sieved sample (0.05 g) was added to 1.5 mL of 80% ethanol. The mixture was then vortex-shaken and mixed. Subsequently, the mixture was placed in a 50 °C water bath for 20 min. The mixture was allowed to cool, and then subjected to 12,000 rpm centrifugation for 10 min. The precipitate was removed. Add 1 mL of 80% ethanol to the precipitate and mix it with shaking for 2 min. Then, put it in a 50 °C water bath for 20 min. Next, cool it, centrifuge it at 12,000 rpm for 10 min, discard the liquid part, leave the solid part, and dry the solid part at 95 °C. After that, add 0.1 mL of a 30% acetyl bromide solution, mix well, and place in a water bath at 60 °C for 1 h. After that, add 0.4 mL of 2 mol/L NaOH solution and 0.5 mL of ice-acetic acid solution, mix well, centrifuged at 12,000 rpm for 3 min, take the supernatant, put into the ultraviolet spectrophotometer in the 280 nm colorimetry (Johnson et al., 1961).

#### 2.3.3 Cellulose measurement

A dried sample (0.05 g) was weighed and placed in a 2-mL centrifuge tube. Then, 1 mL of 60% H_2_SO_4_ was added to the tube, and it was digested in an ice bath for 30 min. The tube was spun at 10,000 rpm for 3 min, and the liquid on top was collected. Dilute the supernatant 50 times, add 30 μL of 2% anthrone to 120 μL of the solution to be measured. Add 250 μL of concentrated H_2_SO_4_ along the wall of the tube. Mix well, and then immediately put it into a boiling water bath for 10 min, take it out and cool it down. Pipette 200 μL into a 96-well enzyme plate, and then measure the absorbance at 620 nm.

#### 2.3.4 Hemicellulose measurement

Hemicellulose assay. The sample was sieved and weighed 0.05 g, added to 1 mL of calcium nitrate solution, boiled water bath for 10 min, centrifuged at 10,000 rpm for 3 min and the precipitate was left. The precipitate was rinsed 3 times with hot distilled water and centrifuged at 10,000 rpm to leave the precipitate. Add 500 μL hydrochloric acid solution (2 mol/L), boil water bath for 1 h, add 500 μL sodium hydroxide solution (2 mol/L), centrifuge at 10,000 rpm for 3 min, aspirate 50 uL of supernatant and add 150 µL of DNS reagent to mix well and then boil water bath for 5 min, cool down and then add 800 μL of distilled water to add 200 μL of supernatant to enzyme plate after mixing well and then aspirate 200 μL of supernatant to the enzyme plate, and then measure the absorbance at 540 nm. Absorbance was measured at 540 nm (Xiong et al., 2005).

### 2.4 Isolation of volatiles by HS-SPME

The extraction of the FCT volatile compounds was carried out by the HS-SPME method using a 50/30 μm DVB/Carboxen/PDMS fiber StableFlex/SS (1 cm) (Sigma Aldrich Supelco). 0.3 ± 0.01 g of tobacco and 6 μL of internal standard deuterated hexanol-D13 at a concentration of 0.948 mg mL^−1^ (dissolved in dichloromethane) were added to a 20 mL headspace flask. The equilibrium time was set at 20 min, and the equilibrium temperature was set at 60 °C in the agitator of Thermo Scientific TriPlus RSH (Thermo Fisher Scientific, Waltham, MA, United States). After the equilibrium, the volatile compounds were adsorbed at the top of the vial by inserting the SPME fiber. The adsorption time was 30 min. After the adsorption, the SPME fiber was desorbed for 5 min while maintaining the GC-MS injection port at 250 °C.

### 2.5 GC–MS analysis

GC–MS analysis was performed using a Thermo Fisher Trace 1,300 gas chromatograph combined with a Thermo Fisher mass spectrometer (both Thermo Fisher Scientific, Waltham, MA). Separation was performed using a TG-Wax column (30 m × 0.25 mm × 0.25 μm; Thermo Fisher Scientific). The chromatographic conditions of helium as the carrier gas were as follows: column flow rate of 1.2 mL/min, injection temperature, transmission line and ion source temperature of 250 °C, 240 °C and 250 °C, respectively. The column heating program was maintained at 50 °C for 2 min, then increased to 120 °C at a rate of 5 °C/min and kept for 4 min, then increased to 200 °C at a rate of 4 °C/min and then increased to 230 °C at a rate of 8 °C/min and kept for 4 min.

The electron ionization (EI) mode was used. The electron energy was −70 eV; the mass spectrometry data were collected in full scan mode with a mass range of m/z 40∼350 and a solvent delay time of 3 min.

### 2.6 Data statistics and analysis

The results of peak resolution were matched with the mass spectral information in the database of the National Institute of Standards and Technology (NIST, v1.6). The retention indices of the compounds were calculated according to the retention time of n-alkane, and then compared with the retention indices in the database (NIST Chemistry WebBook) to verify the results of the characterization. Finally, the relative quantification of the compounds was performed based on the ratio of the peak areas of the characterized compounds to the peak areas of the internal standards. The data were then subjected to principal component analysis (PCA), orthogonal partial least squares discriminant analysis (OPLS-DA) using SIMCA-P version 13.0 software package (Umetrics, Umea, Sweden). Bar stacking plots were generated using Origin. The data were analyzed to generate heat maps using TBtools. LR is a linear model used to solve classification problems, especially binary classification problems. It is one of the most basic, commonly used, and important algorithms in machine learning. The Supplementary Code SA1, developed with the assistance of ChatGPT, is provided in the appendices. Odor Activity Value (OAV) was calculated by the following expression: OAV = Ci/OTi, where the Ci is the concentration and the OTi is the threshold value of the target aromatic components from the book (Gemert, 2025) and previous literature (Liu, 2021). The OAVs are frequently used to evaluate the aromatic compounds. The compound with the OAV>1 is significant for the aroma characteristics. IBM SPSS Statistics was used to perform analysis of ANOVA and *t*-test (P < 0.05) for significance assessment.

## 3 Results and discussion

### 3.1 Analysis of volatile components by HS-SPME-GC-MS

A total of 28 different origins of FCT were analyzed after removal of plasticizers and low-match volatiles. The analysis revealed that all samples possessed the same types of volatile compounds, with variations occurring solely in their composition. A total of 67 volatile components were identified, which belonged to 8 major groups of volatile compounds, including 9 alcohols, 4 phenols, 6 aldehydes, 6 acids, 24 ketones (including four isomers of megastigmatrienone), 2 olefins, 8 heterocycles, and 8 esters (Supplementary Tables SA1, SA2). The nine most abundant compounds in all samples were nicotine, neophytadiene, menthol, acetic acid, benzyl alcohol, nicotyrine, 6,8-Nonadien-2-one, 8-methyl-5-(1-methylethyl) -, (E)-, phenethyl alcohol, and 2-ethylhexanol. Nicotine was the most abundant volatile constituent in all samples, accounting for more than 40% in all samples. Nicotine, a toxic and addictive volatile compound present in tobacco, is characterized by its pungent odor. It is important to note that the detected nicotine content may be inaccurate due to the large peak area in the chromatogram. The more accurate nicotine content would require a separate measurement with a reduced injection volume. Neophytadiene was the second most abundant volatile component in all samples, with a content of more than 14% in all samples. Neophytadiene is a non-pigmented terpene in tobacco, formed by the degradation of chlorophyll during maturation and modulation to form chlorohydrin and then dehydrated. Neophytadiene has a clear aroma and is less irritating, and is itself a precursor of low molecular mass aroma components (Shi et al., 2011). It is evident that neophytadiene confers a distinctive contribution to the aroma of tobacco. The two most abundant volatile components accounted for more than 50% of the total content. The categorization of the volatile constituents with higher contents revealed the presence of benzyl alcohol and menthol among the alcohols, phenol among the phenols, benzaldehyde among the aldehydes, acetic acid among the acids, 6,8-nonadien-2-one, 8-methyl-5- (1-methylethyl)-, (E)- and megastigmatrienone among the ketones, nicotine and nicotyrine among the heterocycles, and 2-ethylhexyl acetate among the esters.

**FIGURE 1:**
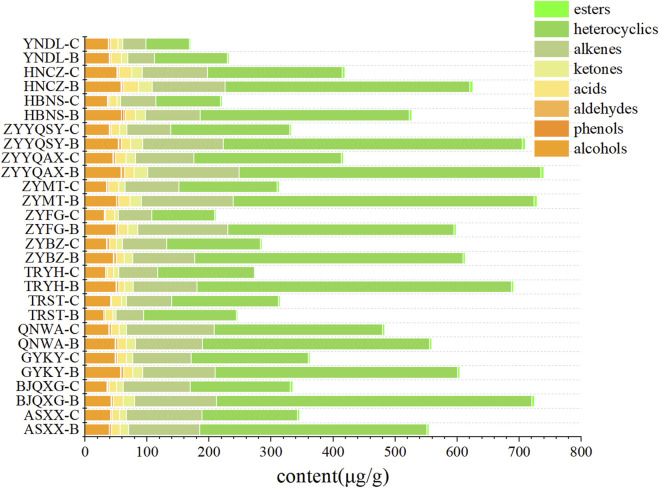
Bar stacking plots of eight types of volatile compounds from different origins.

**FIGURE 2:**
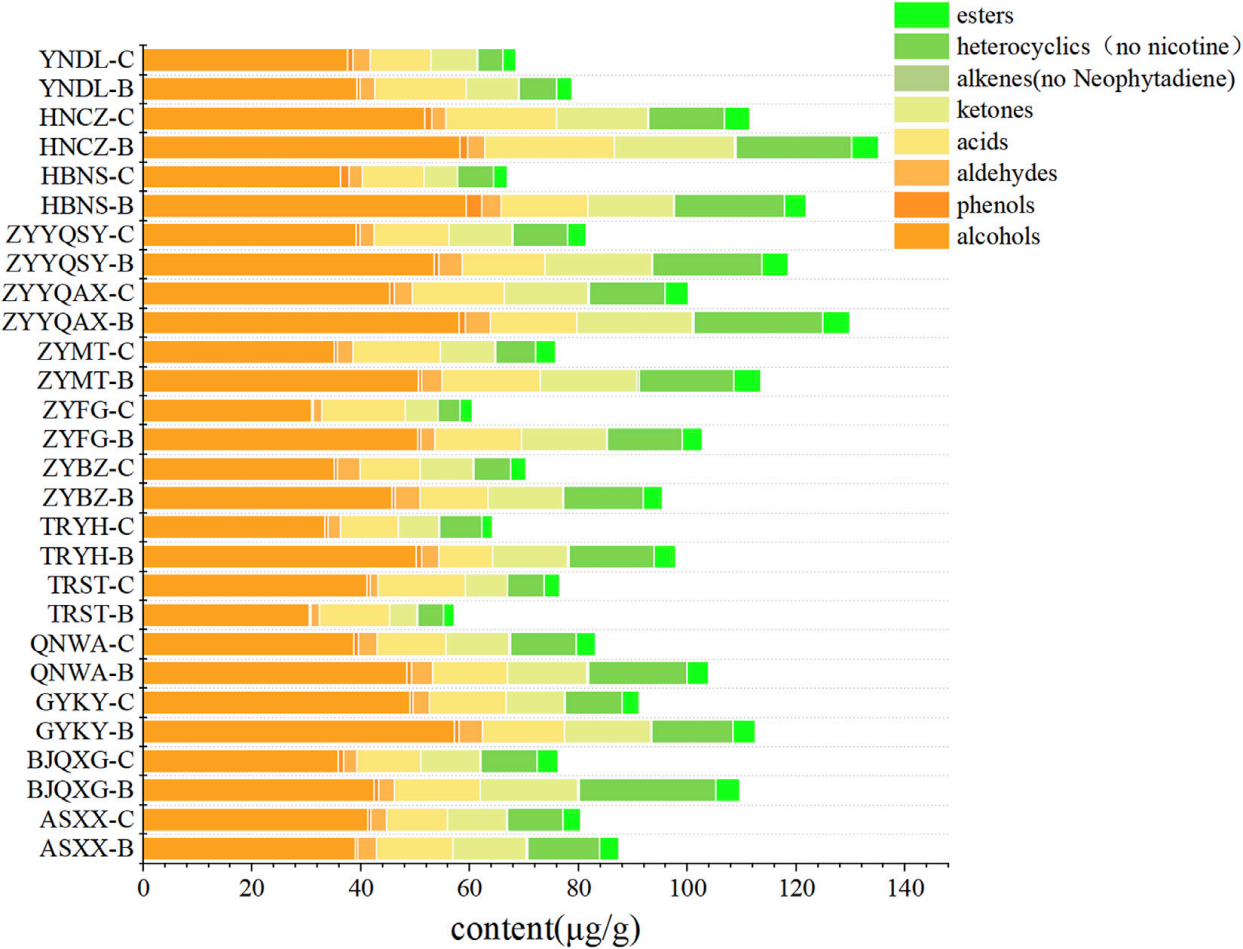
Bar stacking plots of eight types of volatile compounds from different origins (Exclusion of nicotine and neophytadiene).

As demonstrated in Figure 1, the highest levels of volatile compounds in grade B were identified in ZYYQAX, ZYMT, and BJQXG, while the lowest levels were found in YNDL, TRST, and HBNS. The highest levels of volatile compounds in grade C were found in QNWA, HNCZ, and ZYYQAX, and the lowest levels were found in YNDL, HBNS, and ZYFG. The levels of volatile substances in ZYYQAX were generally high, while HBNS had generally low levels of volatile compounds. With the exception of TRST samples, all other samples exhibited higher levels of volatile substances in grade B compared to grade C. The predominant volatile compounds in all samples were identified as heterocyclic and olefinic compounds, with nicotine being the predominant compound in heterocyclic and only two olefinic compounds, primarily contributed by neophytadiene, both of which accounted for more than 90% of the content in their respective categories. To visualize the compounds with lower content, after excluding nicotine and neophytadiene (Figure 2), the content of volatiles detected was higher in class B than in class C for all samples except those with TRST origin. The most abundant types of compounds were alcohols, with the highest levels of menthol and benzyl alcohol. Menthol has cooling, anesthetic and analgesic properties that moderate the irritation and pungency of tobacco. Due to its physiological effects, menthol contributes to the organoleptic quality of smoke and influences the preference of tobacco (Yerger and McCandless, 2011). The most diverse group was ketones, containing four conformations of megastigmatrienone. However, ketones were not among the top three classes of compounds in terms of content, and the relative content was not high. The samples with the highest content of volatiles in the grade B -samples were HNCZ, ZYYQAX, and HBNS, and those with the lowest content were YNDL, ASXXH, and TRST. The samples with the highest content of volatiles in the grade C samples were HNCZ, ZYYQAX and GYKY, and the lowest contents were TRYH, ZYFG and HNNS. It is notable that HNCZ and ZYYQAX exhibited higher concentrations of volatile compounds. The distinction between the two grades lies in the specific leaf region: grade B is designated for the upper leaf, while grade C is allocated for the middle leaf. The *t*-test was used to divide the data into two groups based on grade. There were significant differences in volatile substance content between the two groups. The differences in alcohols, ketones, heterocyclics, heterocyclics without nicotine, and alkenes without neophytadiene were significant. This discrepancy may be attributed to the differing levels of light exposure between the upper and middle leaves within a single plant, which may result in greater accumulation of volatile substances in the upper leaf. Light intensity and quality significantly influence the biosynthesis of secondary metabolites, which are precursors to many volatile aroma compounds (Zhang et al., 2021). However, the presence of more volatile substances does not necessarily guarantee a more significant contribution of aroma substances. The *t*-test was used to divide the data into Guizhou and non-Guizhou categories. There was no significant difference between the two categories of volatile substances.

**FIGURE 3:**
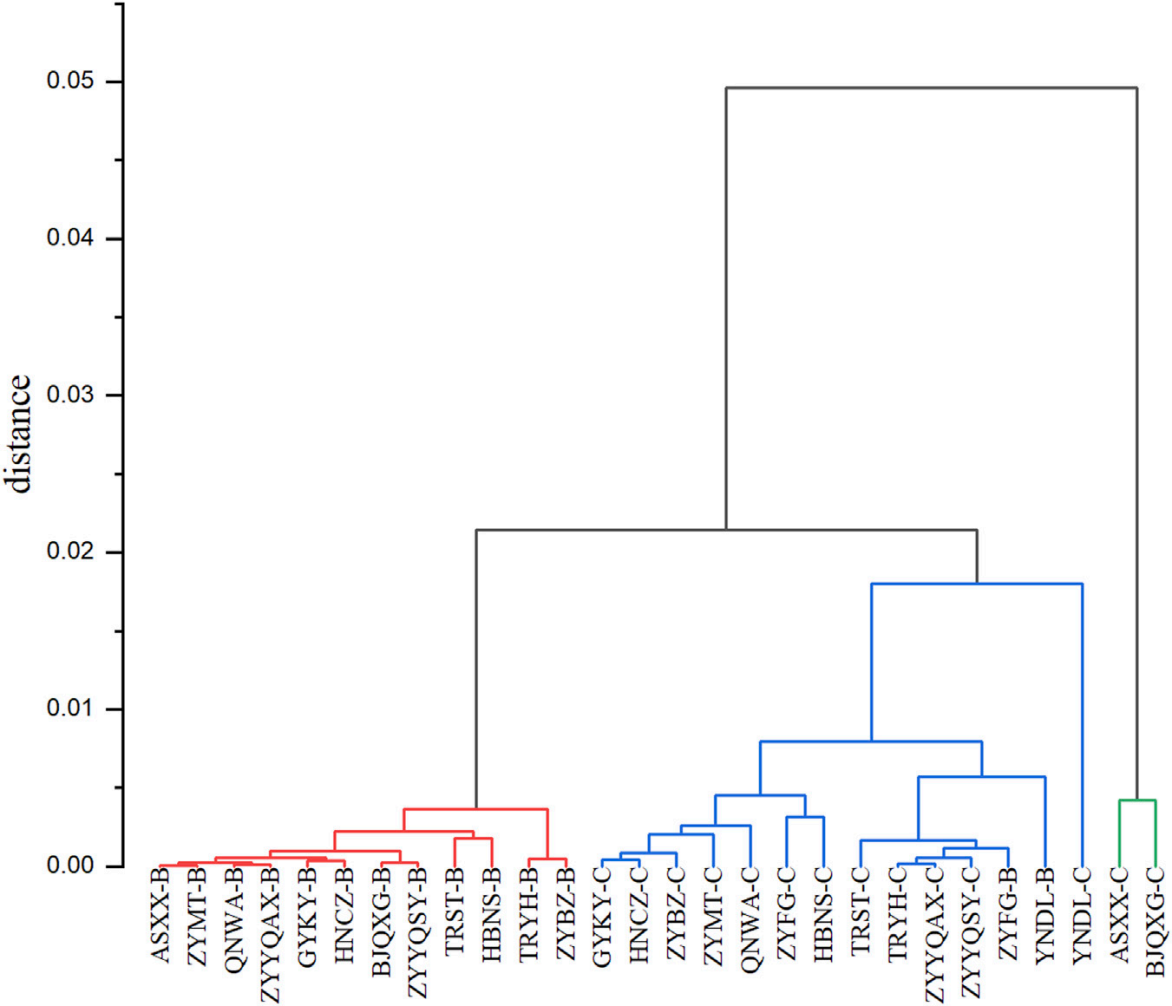
Systematic clustering of different origins.

As demonstrated in the systematic clustering diagram (Figure 3), all the samples are divided into three classes, of which ASXX-C and BJQXG-C are distinct from all the samples of the clustering. The red clustering part of all the grade B samples. The blue clustering part of the samples is mainly grade C samples. The vertical axis represents the distance between the different clusters, which are relatively small. This suggests that the clustering of different samples was also small, and that the systematic clustering can differentiate to a certain degree the levels of the tobacco origin. However, the origin cannot be effectively distinguished.

**FIGURE 4:**
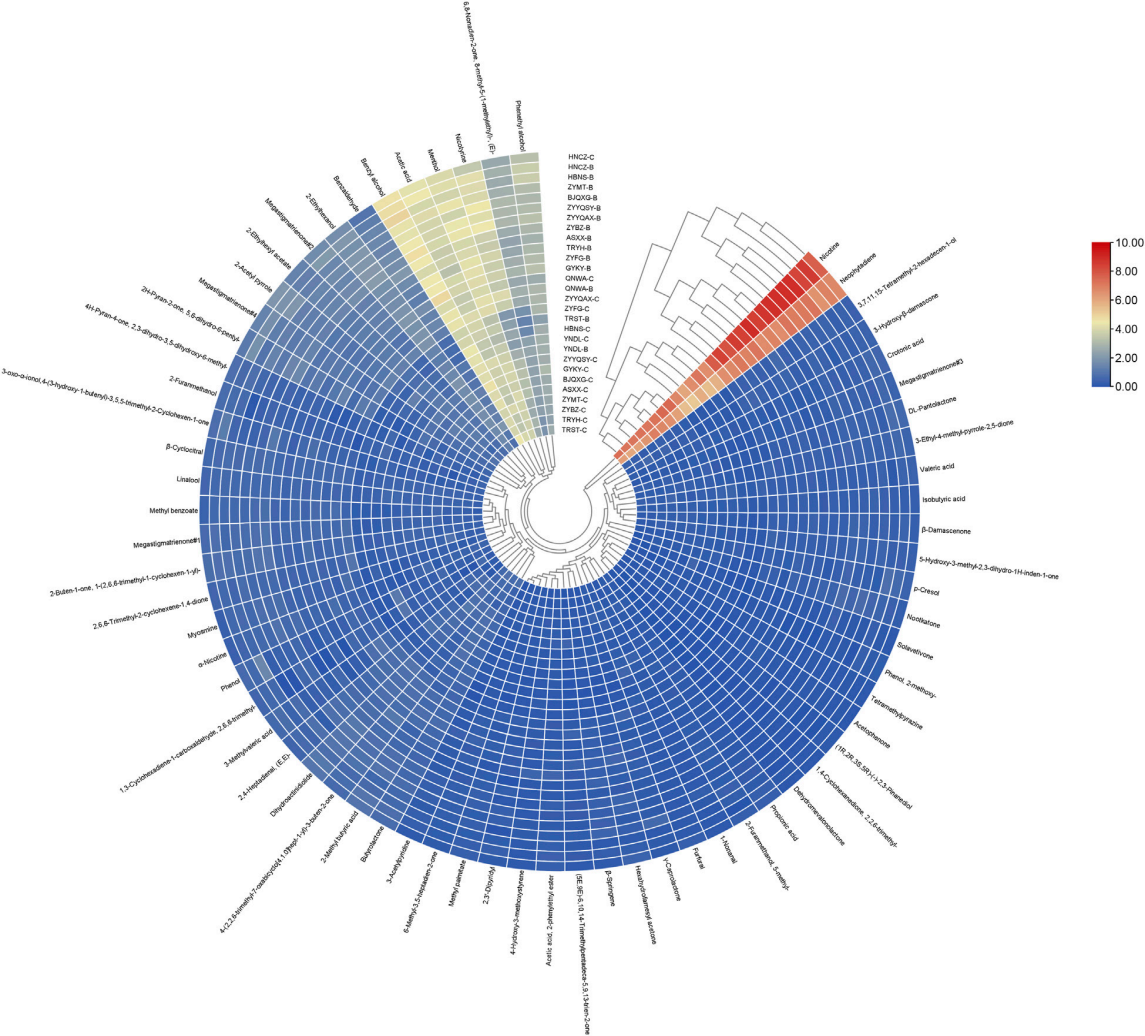
Clustered heat map of the content of all volatiles from different origins.

To facilitate a more thorough investigation of the effect of different compounds on clustering, a clustering heat map was produced (Figure 4). The analysis revealed a consistent overall trend in the content of all volatile compounds across all origins, with minor variations in the content of volatile components between origins. From the standpoint of cluster analysis, nicotine and neophytadiene exhibited a tendency to congregate, likely attributable to their preponderance in abundance relative to other compounds. The second most abundant clusters comprised phenethyl alcohol, nicotyrine, menthol, acetic acid, benzyl alcohol and 6,8-nonadien-2-one, 8-methyl-5-(1-methylethyl)-, (E)-, respectively. Nicotyrine has a pungent odor and is a volatile with a negative correlation to sensory quality (Chen et al., 2021a). Nicotyrine has been reported to be a metabolite of nicotine, is a minor tobacco alkaloid (Jalili et al., 2020). Phenethyl alcohol has a typical rose-like odor, benzyl alcohol has a slight almond flavor, all these odors may be directly related to the honey-sweet scent. The third most abundant clusters were seven compounds with two conformations of megastigmatrienone, respectively. The third most abundant clusters were benzaldehyde, 2-ethylhexanol, megastigmatrienone#1, 2-acetyl pyrrole, 2-ethylhexyl acetate, megastigmatrienone#2, and 2H-pyran-2-one,5,6-dihydro-6-pentyl-. The differences characteristics of these compounds led to the categorization of the different origins into distinct clusters. YNDL-C, YNDL-B, HBNS-C, ZYFG-C, TRST-B, and TRST-C were grouped into a single cluster with the other 22 origins, while the nine origins near the center were divided into three clusters, which were different from the rest of the 19 origins. At the same time, these nine origins contained the roasted cigarettes from all six non-Guizhou producing regions. The FCT samples in this study originated from all nine regions, excluding those from Guizhou. These clusters demonstrated that the heat map clustering of volatile compounds content can differentiate between FCT from different origins. Furthermore, it was established that FCT from the Guizhou production area is distinguishable from other origins with regard to volatile compounds content.

**FIGURE 5:**
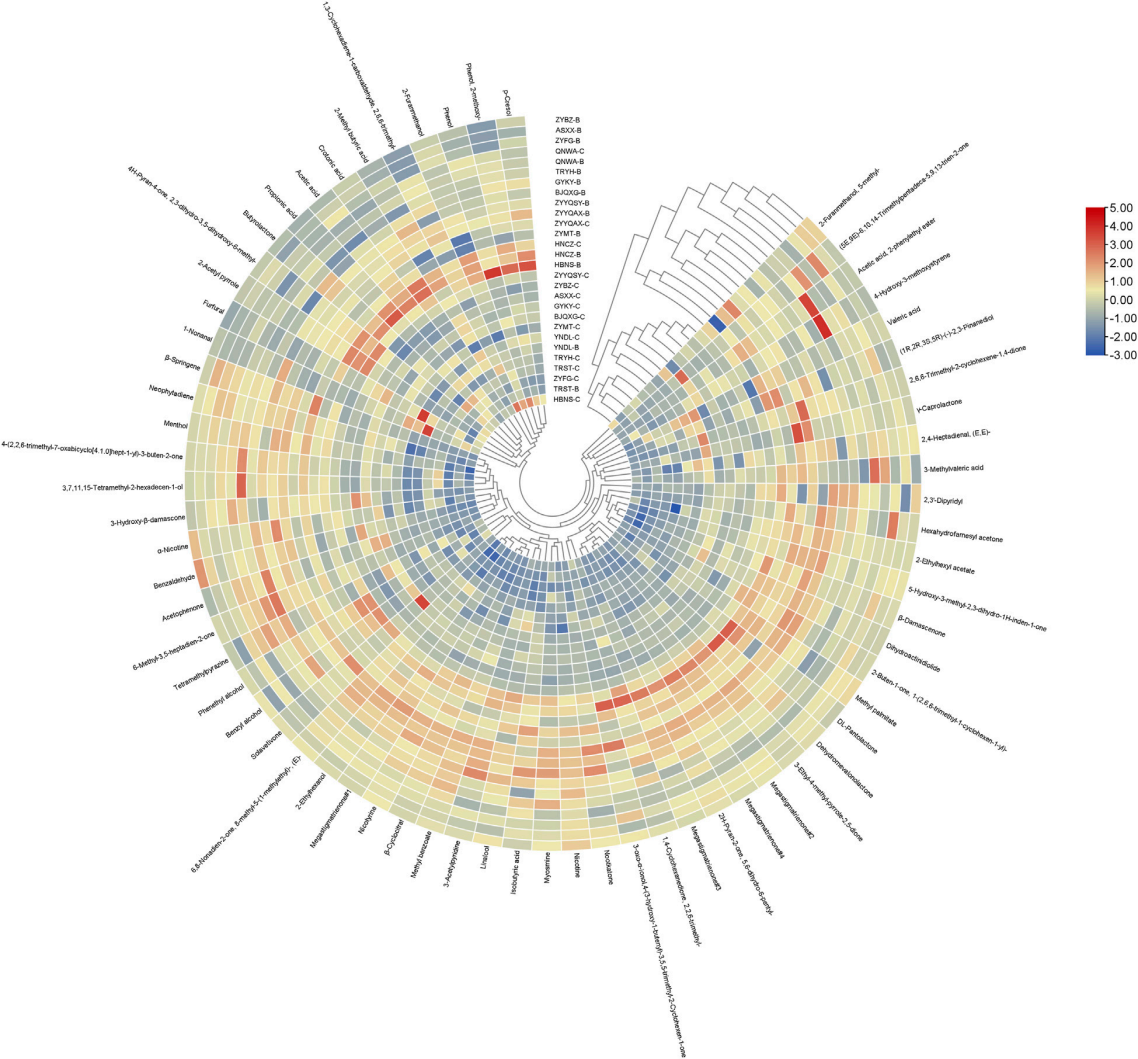
Clustered heat map of the content of all volatiles from different origins with standardized data.

A standardized heatmap (Figure 5) of the data was used shows that all samples were divided into two large clusters, one dominated by Grade C samples and the other dominated by Grade B samples. Despite the possibility of division into two broad categories, the application of heat maps to visualize compounds which differ significantly in impact proves to be a challenging task. This limitation precludes the establishment of a more valid judgment, underscoring the necessity for a more efficacious treatment.

### 3.2 Data science analysis of volatile substances

**FIGURE 6:**
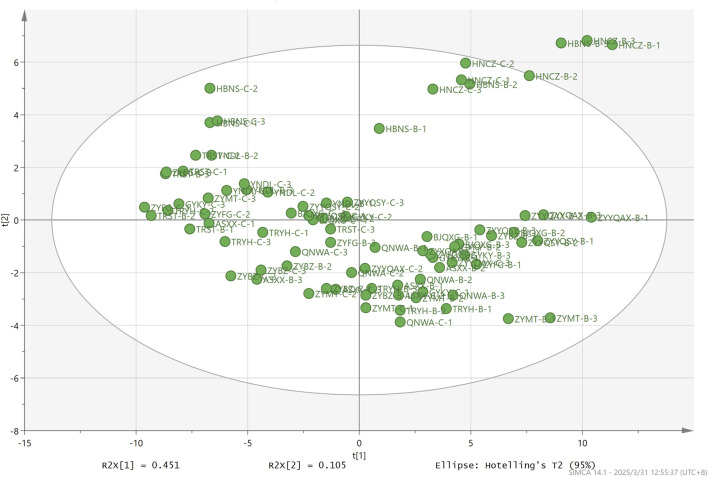
PCA based on volatile of FCT from different origins.

To comprehensively investigate the role and contribution of volatile compounds to the differentiation of origin and grade, a more effective chemometrics and machine learning approach was employed. Principal component analysis (PCA) was an unsupervised learning approach to automatically determine the differences between different groups based on the data. As demonstrated in Figure 6, the initial two principal components analyzed by PCA accounted for 45.1% and 10.5% of the total variance. HNCZ and HBNS, respectively, were distinctly differentiated from the samples of other origins in the secondary principal component. Megastigmatrienone#3, megastigmatrienone#4, β-cyclocitral, 3-ethyl-4-methyl-pyrrole-2,5-dione and 6,8-nonadien-2-one, 8-methyl-5-(1-methylethyl)-, (*E*)-, which are robustly captured by the model with significant information contribution with both R2VX and Q2VX greater than 0.9. It has been established that megastigmatrienone#3, #4 and β-cyclocitral are all degradation products of carotenoids in tobacco. These are produced by the continuous degradation of carotenoids during maturation and mellowing of tobacco leaves. Carotenoids represent a significant category of terpenoids in tobacco, with numerous degradation products playing a crucial role in shaping the distinctive aroma of tobacco (Shi et al., 2011). The findings of the study indicate that PCA is unable to establish a complete correlation between the origin of the samples and the volatile compounds. However, it does demonstrate the capacity to differentiate between several samples that are not from Guizhou province. This finding lends support to the fact that, in general, the volatile compounds of tobacco from Guizhou province can be distinguished from the volatile compounds of tobacco from other origins. In summary, the PCA-based screening of the most contributing compounds is not a useful technique for distinguishing the origin of tobacco.

**FIGURE 7:**
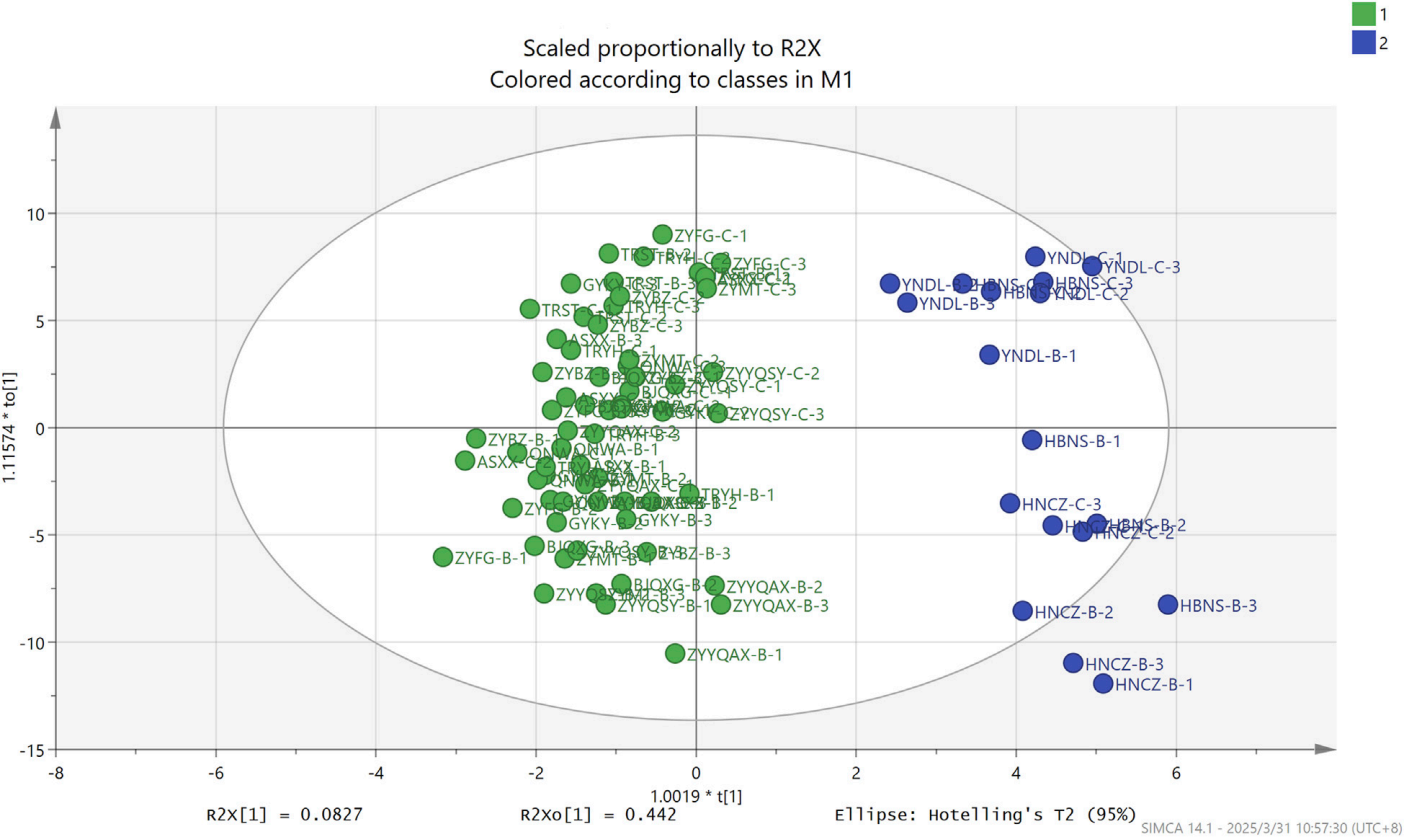
OPLS-DA based on volatile of FCT from different origins.

**FIGURE 8:**
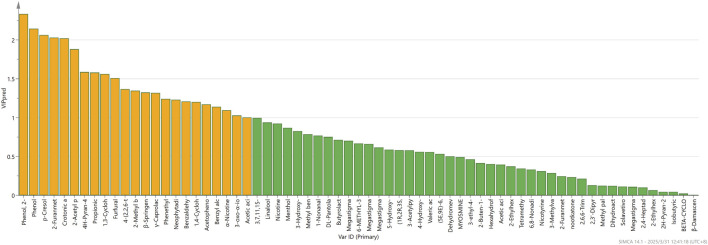
VIP based on volatile of FCT from different origins.

OPLS-DA, a supervised learning approach, facilitates the distinction between different categories determined by which factors. As illustrated in Figure 7 through the OPLS-DA analysis, it can effectively differentiate tobacco from different origins. In this analysis, the independent variable fitting index R2X is 0.617, the dependent variable fitting index R2Y is 0.882, and the model prediction index Q2 is 0.786. R2 and Q2 are greater than 0.5, indicating that the model fitting results are acceptable. The findings suggest that the model demonstrates a high level of variance explained (R2Y) and exhibits robust cross-validated predictive capability (Q2). Furthermore, cross-validation was executed for 200 iterations, and a low intercept (R2 = 0.337 and Q2 = −0.494) substantiates the absence of overfitting in the model. As demonstrated by the VIP (variable importance in projection) projection plot (Figure 8), 23 compounds exhibited VIP values greater than 1 and *p* < 0.05, while five substances (phenol, 2-methoxy-, phenol, p-Cresol, 2-furanmethanol, and crotonic acid) demonstrated VIP values greater than 2. These compounds can be utilized as differentiating indicators among honey-sweet flavored and other aroma tobacco types.

Machine learning is predicated on numerous classification models that are frequently employed to construct hyperplanes, curves and other such functions for the purpose of classification. These models are more mature and have a wider range of applications in the field of volatiles (Ji et al., 2023; Zhong et al., 2024). The LR model was applied in this study, here are the 5-fold cross-validation results: accuracy = 0.965, recall = 1, precision = 0.963, F1-score = 0.979, AUC (area under the curve) = 1. The logistic regression model demonstrated high predictive accuracy with complete class separation. LR has better differentiation effect than OPLS-DA, which can effectively differentiate the samples from Guizhou and non-Guizhou provinces. The effect is better, it may be due to the small amount of data itself, which is an easier task for machine learning. The feature importance was then ranked, and the ten compounds with the highest contribution were identified as phenol, 2-methoxy-, 1-nonanal, phenol, furfural, crotonic acid, 1,3-cyclohexadiene-1-carboxaldehyde, 2,6,6-trimethyl-1,3-cyclohexadiene-1-carboxaldehyde (safranal), p-cresol, 2-furanmethanol, 2-acetyl pyrrole and neophytadiene. The volatiles 2-furanmethanol, 2-acetylpyrrole, and neophytadiene are likely responsible for the honey-sweet flavored of FCT in Guizhou, which differs from the aroma of tobacco from other regions. These volatiles are the key factors contributing to the sweet aroma of FCT in Guizhou. Phenol, 2-methoxy- in plants has been reported to be a degradation product of lignin (Zhang et al., 2018). The aroma is smoky and slightly sweet, with a stress and anxiety-relieving effect (Takemoto et al., 2022). Phenol, 2-methoxy-, a major aroma compound in plant samples that have been heated above 40 °C, such as barley and coffee (Joung et al., 2018; Ochiai et al., 2014). The smokiness and sweetness of the aroma may have a direct influence on its overall honey-sweetness. P-Cresol and phenol have a distinctive phenolic aroma and a pungent odor. Furfural has a sweet, toasty aroma that is significantly more affected by climate than the other volatiles (Deng et al., 2010). Collectively, these findings suggest that furfural is an important volatile compound for distinguishing origin and type of aroma. 1,3-Cyclohexadiene-1-carboxaldehyde, 2,6,6-trimethyl-also known as safranal, has a tobacco aroma and pungent flavor. 2-Furanmethanol has also been reported as an important volatile compound in other honey-sweet flavored related studies (Li et al., 2024). 2-Furanmethanol exerts a substantial influence on sensory quality (Song et al., 2012). The analysis revealed the presence of phenol, 2-methoxy-, p-cresol, 2-furanmethanol, crotonic acid, phenol, safranal, and furfural in both statistical methods. These compounds are regarded as key volatile compounds, as they are employed to differentiate between origin and aroma. Furthermore, these compounds are believed to be the volatile compounds that are most likely to influence the differentiation of Guizhou honey-sweet tobacco from other aroma types. The results of system clustering demonstrate that system clustering is incapable of distinguishing between sources. This approach is more influenced by the actual content of the compounds themselves, as the distinction is made based on the differences in compound content between grades B and C. OPLS-DA and LR handle the effects of compound types and content more reasonably, enabling better differentiation between the two. Furthermore, OPLS-DA and LR are supervised learning methods that provide labels in advance during data analysis, allowing for the selection of dimensions that aid in differentiation. PCA, as an unsupervised learning method, also yields relatively average results. The employment of compound content and types to differentiate between Guizhou origins has been demonstrated to yield superior outcomes when utilizing supervised learning methodologies.

### 3.3 OAV to identify key aroma compounds for differentiating honey-sweet flavored tobaccos

Statistical analysis was employed to identify key volatiles that could be included in honey-sweet flavored FCT in Guizhou, which is different from other producing regions. 23 of them were selected from OPLS-DA and the 10 with the highest contribution from the machine learning model, for a total of 27 volatile compounds screened by all methods. To identify the key aroma substances among them, the psychophysics of olfaction was combined with the concentration and odor threshold of the compounds to determine the compounds that contribute to the overall aroma profile. OAV is a reliable parameter that can be used to assess the contribution of a single compound to the overall aroma of a plant. Volatile compounds with an OAV > 1 are usually considered to be aroma-active compounds that contribute significantly to the overall aroma. In the present study, OAV was applied to quantitatively analyze the key aroma compounds that distinguish Guizhou honey-sweet flavored tobacco from other aroma types.

The analysis revealed a total of nine compounds with OAVs greater than 1 (Supplementary Table SA3). 1-nonanal had the highest OAV, which has a greenish, slightly sweet, slightly beeswax floral aroma, and 1-nonanal is one of the major volatile compounds in cigar tobaccos, where the main flavors are sweet, light and grassy, with more emphasis on fermentation (Wu et al., 2023). 1-Nonanal, a volatile substance, has been shown to have a higher correlation with tobacco pests. This correlation may ultimately affect the flavor of FCT indirectly. However, the relationship between these two substances has not yet been reported (Li et al., 2020; Liu Z. et al., 2022; Sun et al., 2012). The fungal community *s__Penicillium_vulpinum* showed a significant positive correlation with Nonana (Wu et al., 2023). The second highest OAV is β-cyclocitral, a chemical compound with a floral and fruity aroma. It is the main volatile compound in pineapple nectar, which is characterized by a pronounced honey-sweet flavored (Barros-Castillo et al., 2022). This may result in a close relationship with honey-sweet FCT. Benzaldehyde has an OAV of less than 1 only in ZYFG-C and HBNS-C. γ-Caprolactone has an OAV greater than 1 only in ZYMT-B, ZYMT-C, and ZYYQAX-C. 6,8-Nonadien-2-one, 8-(methyl-5-(1-methylethyl)-, (E)- is only present in TRST-B. Phenol, 2-methoxy- has an OAV of less than 1 only in TRST-B. Phenol, 2-methoxy- has an OAV less than 1 only in ZYMT-C. Phenol, 2-methoxy- has an OAV less than 1 only in ZYYQAX-C. The compounds of these origins correspond to OAVs that are different from those of other origins. The present study examines the correlation between disparate origins and diverse aroma types. In this context, OAV = 1 is important as a threshold value, and there is an effect of subthreshold olfaction on blends of odors. Furthermore, subthreshold olfaction has been shown to have an effect on sweetness (Labbe et al., 2007). The key aroma compounds that distinguish Guizhou-cured tobacco from other tobaccos in terms of honey-sweet flavored are 1-Nonanal, Benzaldehyde, β-cyclocitral, 6,8-Nonadien-2-one, 8-methyl-5-(1-methylethyl)-, (E)-, Phenol, 2-methoxy, Benzyl alcohol, Phenethyl alcohol, p-Cresol. The aroma of tobacco is the result of a combination of internal factors, such as genetics and botany, and external factors, such as ecology, cultivation, physiological and biochemical metabolism, synthesis and degradation of precursors, roasting and microorganisms (Chen et al., 2021b; Gui et al., 2024; Li et al., 2022; Zhang et al., 2024; Zhao et al., 2024; Zhengfeng et al., 2024; Zou et al., 2023).

### 3.4 Analysis of cell wall components

**FIGURE 9:**
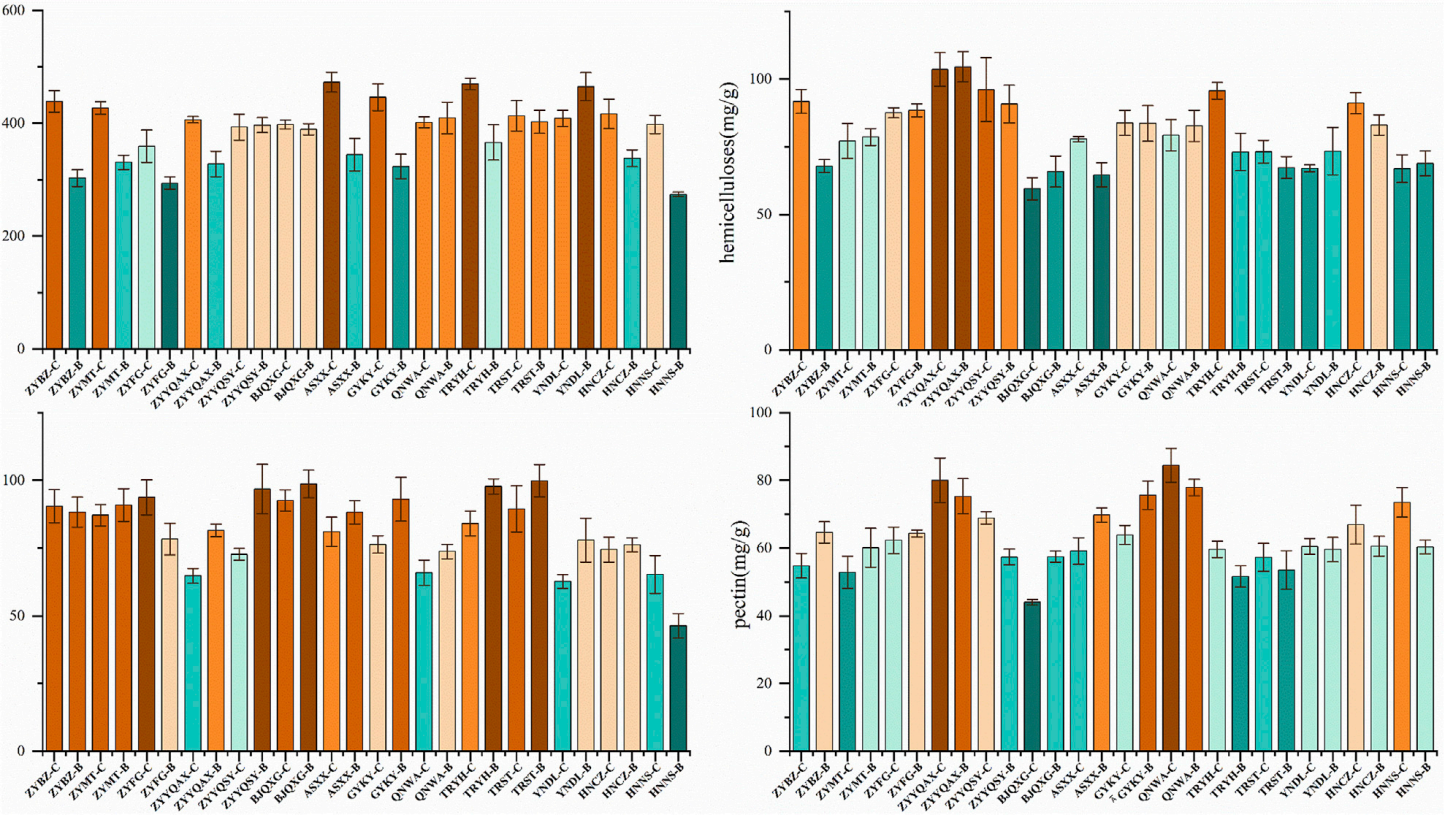
CWC content of FCT from different origins.

The plant cell wall is a network of interacting polysaccharides, proteins, small polyphenol molecules and water, and the main load-bearing element is cellulose, which is embedded in a mixture of hemicellulose, pectin and lignin (Pedersen et al., 2023). As can be seen in Figure 9, the application of ANOVA revealed that there is an obvious pattern of the four CWCs (Supplementary Table 1). The samples with the highest cellulose content are ASXX-C, TRYH-C and YNDL-B, and the lowest are ZYBZ-B, ZTFG-B and HNNS-B. The samples with the highest hemicellulose content are ZYYQAX-C, ZYYQAX-B, and ZYBZ-C, and the lowest are BJQXG-C, BJQXG-B, and ASXX-B. The highest levels of lignin were observed in ZYYQSY-B, BJQXG-B, and TRYH-B, while the lowest levels were detected in YNDL-B, HBNS, and ZYYQAX-C. Conversely, the highest pectin contents were observed in ZYYQAX-C, QNWA-C, and QNWA-B, while the lowest contents were found in BJQXG-C and TRYH-B. The cellulose content of the vast majority of origins is higher for grade B than for grade C. Conversely, QNWA and YNDL exhibited higher lignin content compared to grade B. ZYYMT, ZYYQAX, and HNNS showed the opposite trend. No discernible patterns were observed for hemicellulose and pectin. The factors influencing the quantity of CWC include genes, growth and development, and origin, among others (He et al., 2020). In this study, the tobacco varieties are constant, as are the intrinsic factors (e.g., genes), harvesting conditions, and developmental stages of the tobacco leaves. The primary factors that can exert a substantial influence on the content of CWC are the variations in natural environmental conditions between distinct production areas, such as altitude, climate, and other factors. The application of *t*-test revealed that there were no significant differences in pectin and hemicellulose between samples of grades B and C. However, a significant divergence was observed in cellulose and lignin. A notable distinction was observed in the lignin composition of samples originating from the Guizhou region and those from other regions. However, no significant variations were detected in the pectin and cellulose content.

As plants undergo the process of growth, it is required to release the cell walls to allow for expansion, usually by breaking down the binders called pectin, which are acidic polysaccharides containing galacturonic acid residues (Rui et al., 2017). This process includes the degradation of pectin. In a similar manner, cellulose and hemicellulose are macromolecular polysaccharides composed of glucose. The degradation of these polysaccharides produces volatile organic compounds, with aldehydes and ketones being the most common degradation products (Zhai and Edgar, 2024). In this study, ketones were found to be both abundant and significant. It is possible that they are affected by these CWC. Lignin, a hydrophobic polyphenolic compound formed by phenylalanine derivatives, has been shown to be closely related to the degradation of phenolic compounds (Chen et al., 2021a). The most abundant phenolic compound observed in lignin was 2-methoxy-4-methylphenol, eugenol and phenol, 2-methoxy, respectively (Başakçılardan Kabakcı and Tanış, 2021). Phenylalanine derivatives play a major role as aroma precursors and can form polyphenolic secondary metabolites via phenylpropanoids in the flavonoid pathway (Shi et al., 2011). The study identified four phenolic compounds, of which phenol, 2-methoxy, phenol and p-cresol are important aroma compounds. Furthermore, it has been established that phenylalanine can undergo degradation via the STREKER pathway, resulting in the formation of phenylacetaldehyde and subsequently phenethyl alcohol. The latter is recognized as a critical aroma compounds within tobacco (Song et al., 2012). The significant differences in lignin content suggest that these related volatile compounds are more likely to be responsible for the characteristic aroma of Guizhou FCT, which warrants further validation and research.

## 4 Conclusion

In this study, the HS-SPME/GC-MS technique was employed to evaluate the volatile compounds of tobaccos from 14 origins, each containing upper leaf (Grade B) and middle leaf (Grade C), which can be categorized into Guizhou producing regions and non-Guizhou producing regions. The analysis yielded the identification of 27 volatile compounds that distinguished Guizhou honey-sweet flavored FCT from other tobaccos through the application of data science methodologies. The OAV of molecular sensory science identified eight key aroma active substances: 1-nonanal, benzaldehyde, β-cyclocitral, 6,8-nonadien-2-one, 8-methyl-5-(1-methylethyl)-, (E)-phenol, 2-methoxy-, benzyl alcohol, phenethyl alcohol, p-cresol, which are the key aroma compounds to distinguish Guizhou FCT from other tobaccos, and the most important ones are β-cyclocitral and 1-nonanal.Guizhou FCT was found to differ significantly from tobacco from other regions in terms of volatile substances, as confirmed by machine learning. The analysis enabled the distinction between Guizhou FCT and FCT from nearby production areas based on volatile substances. Finally, the CWC present in different production areas were detected, including cellulose, hemicellulose, pectin, and lignin, which are precursors of certain volatile substances. The CWC were not effective differentiation indices between different production areas and grades. In summary, the integration of data science and flavoromics with volatile compound data emerges as a promising approach for the identification of the origin and aroma of FCT. This approach facilitates the exploration of variations in volatile matter between different production areas, the reflection of aroma differences across diverse production areas, and the establishment of a foundation for the quality indicators of different aroma tobacco and product research and development.

## Data Availability

The original contributions presented in the study are included in the article/Supplementary Material, further inquiries can be directed to the corresponding authors.
