# Supplementary material for: Decoding honey-sweet flavored flue-cured tobacco from Guizhou with data science and flavoromics by volatile and cell wall components
Source: Front Chem. 2025 Oct 1;13:1613828. doi: 10.3389/fchem.2025.1613828 (PMC12521107; doi:10.3389/fchem.2025.1613828)
Supplement: Supplementary file 1 [file Table2.docx]

| **Table SA.1** content of volatile compounds of B grade (μg/g) | | | | | | | | | | | | | | | | |
| --- | --- | --- | --- | --- | --- | --- | --- | --- | --- | --- | --- | --- | --- | --- | --- | --- |
| NO. | CAS | Compound | ASXX | BJQXG | GYKY | QNWA | TRST | TRYH | ZYBZ | ZYFG | ZYMT | ZYYQAX | ZYYQSY | HBNS | HNCZ | YNDL |
| 1 | 103-09-3 | 2-Ethylhexyl acetate | 1.2661±0.1149 | 1.5365±0.3795 | 1.2701±0.0977 | 1.3247±0.1873 | 0.7154±0.0105 | 1.366±0.1359 | 1.273±0.2747 | 1.2945±0.0692 | 1.7259±0.3828 | 1.7707±0.2568 | 1.7138±0.2727 | 1.4569±0.413 | 1.4933±0.1431 | 1.2235±0.0975 |
| 2 | 124-19-6 | 1-Nonanal | 0.079±0.0219 | 0.1937±0.0883 | 0.083±0.0119 | 0.1017±0.0163 | 0.1547±0.0559 | 0.1036±0.0106 | 0.0911±0.029 | 0.0758±0.0067 | 0.1011±0.0043 | 0.0686±0.0052 | 0.0904±0.0036 | 0.0659±0.0158 | 0.0787±0.0153 | 0.3012±0.0284 |
| 3 | 64-19-7 | Acetic acid | 12.5367±3.1773 | 13.905±0.9069 | 13.4932±0.5103 | 11.5941±0.7127 | 11.8755±3.6788 | 8.897±0.4001 | 11.2182±3.0535 | 14.4135±1.3084 | 16.2006±1.443 | 14.3164±1.3058 | 13.5684±1.2808 | 14.4708±1.6125 | 21.2687±2.4669 | 15.0534±3.1347 |
| 4 | 98-01-1 | Furfural | 0.106±0.0247 | 0.0829±0.0127 | 0.1345±0.0134 | 0.1114±0.0114 | 0.0524±0.0003 | 0.0912±0.0122 | 0.0892±0.0297 | 0.116±0.0067 | 0.1381±0.015 | 0.103±0.0115 | 0.1187±0.0157 | 0.1256±0.0115 | 0.1278±0.0129 | 0.2092±0.0643 |
| 5 | 1124-11-4 | Tetramethylpyrazine | 0.063±0.0112 | 0.0776±0.0209 | 0.1183±0.0045 | 0.1228±0.0196 | 0.0589±0.0059 | 0.1073±0.0152 | 0.0825±0.0241 | 0.1041±0.0074 | 0.0838±0.0129 | 0.106±0.0224 | 0.0847±0.0025 | 0.1042±0.0192 | 0.0702±0.0095 | 0.0672±0.0155 |
| 6 | 104-76-7 | 2-Ethylhexanol | 2.3336±0.4444 | 2.9359±0.2103 | 2.9176±0.3887 | 2.7892±0.1385 | 1.2771±0.0918 | 2.286±0.1034 | 2.498±0.6313 | 2.6734±0.0888 | 3.0766±0.3311 | 3.4369±0.1197 | 3.2513±0.024 | 2.4615±0.4881 | 3.0081±0.2739 | 2.7975±0.1455 |
| 7 | 4313-03-5 | 2,4-Heptadienal, (E,E)- | 0.5992±0.1332 | 0.21±0.0254 | 0.68±0.0499 | 0.7189±0.1475 | 0.2058±0.0324 | 0.4715±0.0238 | 0.6385±0.0835 | 0.5531±0.1076 | 0.7921±0.1368 | 0.7426±0.0777 | 0.7842±0.062 | 0.4364±0.043 | 0.5755±0.0655 | 0.606±0.1402 |
| 8 | 100-52-7 | Benzaldehyde | 2.1242±0.2199 | 1.8014±0.1651 | 2.3519±0.1847 | 1.8683±0.176 | 0.6073±0.0778 | 1.6749±0.0735 | 3.1986±1.1201 | 1.3528±0.1972 | 1.988±0.1919 | 2.3853±0.3928 | 2.1763±0.2399 | 1.7263±0.3403 | 1.3652±0.1627 | 0.9368±0.1882 |
| 9 | 79-09-4 | Propionic acid | 0.1223±0.027 | 0.1604±0.0151 | 0.1039±0.0107 | 0.1348±0.0132 | 0.1631±0.0335 | 0.0914±0.0086 | 0.1147±0.0285 | 0.1173±0.0183 | 0.1599±0.0132 | 0.1394±0.0304 | 0.1473±0.0218 | 0.1526±0.0216 | 0.2607±0.0187 | 0.172±0.0345 |
| 10 | 78-70-6 | Linalool | 0.391±0.0753 | 0.4846±0.0358 | 0.5457±0.0444 | 0.4638±0.0287 | 0.3032±0.0376 | 0.4872±0.0255 | 0.4779±0.1252 | 0.4479±0.0398 | 0.5167±0.0635 | 0.5114±0.0295 | 0.51±0.0088 | 0.569±0.1201 | 0.5239±0.0374 | 0.4449±0.0602 |
| 11 | 79-31-2 | Isobutyric acid | 0.2255±0.0436 | 0.3057±0.0125 | 0.2633±0.0219 | 0.2614±0.0033 | 0.1388±0.0097 | 0.1643±0.0056 | 0.2085±0.0459 | 0.2135±0.0224 | 0.2971±0.0314 | 0.2079±0.015 | 0.2541±0.0107 | 0.2613±0.0319 | 0.2324±0.0215 | 0.1823±0.0331 |
| 12 | 1604-28-0 | 6-Meyhyl-3,5-heptadien-2-one | 0.1563±0.0373 | 0.2064±0.0103 | 0.3015±0.0654 | 0.2149±0.061 | 0.1042±0.0082 | 0.2689±0.0255 | 0.1616±0.0425 | 0.1668±0.0194 | 0.2043±0.0223 | 0.1949±0.0127 | 0.1988±0.0154 | 0.1695±0.0208 | 0.176±0.0075 | 0.1314±0.0305 |
| 13 | 432-25-7 | β-Cyclocitral | 0.4612±0.1047 | 0.6133±0.0287 | 0.5545±0.0288 | 0.5878±0.0469 | 0.2808±0.0452 | 0.5499±0.0125 | 0.4746±0.1278 | 0.4976±0.0567 | 0.6006±0.0445 | 0.6406±0.0254 | 0.6005±0.0147 | 0.6305±0.0805 | 0.5746±0.0872 | 0.3986±0.0596 |
| 14 | 93-58-3 | Methyl benzoate | 0.3988±0.0863 | 0.5294±0.0516 | 0.5989±0.0364 | 0.6183±0.0849 | 0.1705±0.0246 | 0.5648±0.028 | 0.4385±0.1176 | 0.4225±0.0442 | 0.5236±0.0672 | 0.6594±0.0471 | 0.5902±0.024 | 0.533±0.0605 | 0.5167±0.1047 | 0.2208±0.033 |
| 15 | 96-48-0 | Butyrolactone | 0.3907±0.0897 | 0.6213±0.0431 | 0.6381±0.0269 | 0.5274±0.035 | 0.5879±0.0835 | 0.4716±0.0223 | 0.4543±0.1155 | 0.4835±0.0375 | 0.5086±0.0327 | 0.5384±0.0089 | 0.5048±0.0227 | 0.5526±0.0978 | 0.7294±0.05 | 0.4865±0.0778 |
| 16 | 15356-70-4 | Menthol | 14.8471±3.0867 | 12.9308±0.9622 | 16.2196±0.3352 | 17.7452±0.8078 | 10.4285±1.0361 | 18.1517±0.7226 | 14.3153±3.4952 | 17.4151±1.7456 | 19.4617±1.7187 | 19.5097±0.6277 | 20.0969±0.3073 | 13.2676±2.0536 | 16.8505±1.5679 | 12.4343±2.2839 |
| 17 | 116-26-7 | 1,3-Cyclohexadiene-1-carboxaldehyde, 2,6,6-trimethyl- | 0.0235±0.0065 | 0.0372±0.0114 | 0.4789±0.0242 | 0.3988±0.0372 | 0.206±0.029 | 0.3488±0.0194 | 0.0404±0.007 | 0.0255±0.0022 | 0.0355±0.021 | 0.5668±0.0293 | 0.4939±0.0571 | 0.5076±0.0854 | 0.5146±0.036 | 0.2945±0.0339 |
| 18 | 98-86-2 | Acetophenone | 0.0818±0.0195 | 0.1143±0.0146 | 0.1949±0.0428 | 0.1338±0.016 | 0.0333±0.0063 | 0.1168±0.0264 | 0.0659±0.0169 | 0.0944±0.007 | 0.1062±0.0103 | 0.0981±0.0447 | 0.1396±0.0779 | 0.0649±0.0277 | 0.0978±0.0161 | 0.0605±0.0183 |
| 19 | 98-00-0 | 2-Furanmethanol | 0.3371±0.0642 | 0.2918±0.0338 | 0.2957±0.0152 | 0.2938±0.0202 | 0.2994±0.0359 | 0.2898±0.0189 | 0.2994±0.0994 | 0.3559±0.0252 | 0.0685±0.0082 | 0.3989±0.0459 | 0.4139±0.0191 | 0.5721±0.1148 | 0.6148±0.0481 | 0.2976±0.0491 |
| 20 | 116-53-0 | 2-Methyl butyric acid | 0.4723±0.0945 | 0.8856±0.1142 | 0.7436±0.0379 | 0.6217±0.0211 | 0.4117±0.0146 | 0.3807±0.0072 | 0.4899±0.1379 | 0.6114±0.0511 | 0.4446±0.0482 | 0.7373±0.072 | 0.776±0.0219 | 0.5917±0.2193 | 0.9659±0.112 | 0.7441±0.0574 |
| **Table A.1** (continued) | | | | | | | | | | | | | | | | |
| 21 | 1125-21-9 | 2,6,6-Trimethyl-2-cyclohexene-1,4-dione | 0.4321±0.0888 | 0.5275±0.05 | 0.4632±0.0212 | 0.461±0.0455 | 0.197±0.0219 | 0.387±0.0109 | 0.4144±0.089 | 0.4806±0.0669 | 0.6221±0.0679 | 0.5458±0.0213 | 0.5312±0.0215 | 0.456±0.0793 | 0.5362±0.0396 | 0.3838±0.0738 |
| 22 | 695-06-7 | γ-Caprolactone | 0.2017±0.0175 | 0.1581±0.0205 | 0.1969±0.0104 | 0.1821±0.02 | 0.0756±0.0027 | 0.2017±0.0235 | 0.1509±0.0297 | 0.1645±0.0311 | 0.5669±0.0549 | 0.1552±0.019 | 0.1523±0.0025 | 0.0889±0.0067 | 0.1589±0.009 | 0.0624±0.0082 |
| 23 | 3857-25-8 | 2-Furanmethanol, 5-methyl- | 0.2081±0.0279 | 0.1732±0.0212 | 0.1317±0.0085 | 0.1496±0.0091 | 0.0893±0.0064 | 0.1477±0.0111 | 0.2017±0.0114 | 0.1748±0.0038 | 0.2795±0.0474 | 0.1787±0.0245 | 0.1163±0.0083 | 0.2002±0.0724 | 0.0218±0.0047 | 0.1247±0.0049 |
| 24 | 54868-48-3 | 6,8-Nonadien-2-one, 8-methyl-5-(1-methylethyl)-, (E)- | 4.3103±0.8032 | 7.3829±0.3522 | 6.2536±0.0227 | 5.7679±0.6655 | 1.6064±0.15 | 4.4514±0.1839 | 5.0929±1.093 | 5.5681±0.7915 | 5.6917±0.6585 | 6.6996±0.3402 | 6.9992±0.1959 | 5.0475±1.1099 | 5.8315±0.3526 | 4.407±1.0783 |
| 25 | 109-52-4 | Valeric acid | 0.2429±0.0632 | 0.216±0.0107 | 0.2447±0.0168 | 0.2615±0.0141 | 0.1735±0.0197 | 0.2079±0.0101 | 0.253±0.0695 | 0.283±0.0062 | 0.3152±0.0273 | 0.2243±0.0163 | 0.2625±0.0167 | 0.114±0.0319 | 0.3517±0.0236 | 0.3318±0.025 |
| 26 | 20547-99-3 | 1,4-Cyclohexanedione, 2,2,6-trimethyl- | 0.0347±0.006 | 0.0629±0.004 | 0.0329±0.0099 | 0.0363±0.0018 | 0.029±0.0026 | 0.0576±0.0035 | 0.0454±0.0091 | 0.0432±0.0061 | 0.0461±0.0059 | 0.0594±0.0036 | 0.0505±0.0102 | 0.0704±0.0031 | 0.0888±0.0088 | 0.0266±0.0024 |
| 27 | 3724-65-0 | Crotonic acid | 0.2095±0.0424 | 0.2672±0.028 | 0.2183±0.0168 | 0.1878±0.0093 | 0.1667±0.0105 | 0.1445±0.0137 | 0.2061±0.0732 | 0.1761±0.005 | 0.2777±0.043 | 0.2976±0.0865 | 0.2771±0.0157 | 0.322±0.0584 | 0.4789±0.0202 | 0.2651±0.0155 |
| 28 | 105-43-1 | 3-Methylvaleric acid | 0.4847±0.1113 | 0.2507±0.011 | 0.2556±0.0106 | 0.9951±0.0314 | 0.1219±0.0042 | 0.1081±0.0072 | 0.1971±0.0559 | 0.2413±0.0076 | 0.6324±0.0496 | 0.3172±0.0219 | 0.2742±0.0229 | 0.372±0.0402 | 0.6416±0.0487 | 0.2583±0.0224 |
| 29 | 35044-68-9 | 2-Buten-1-one, 1-(2,6,6-trimethyl-1-cyclohexen-1-yl)- | 0.4678±0.1017 | 0.677±0.0391 | 0.4408±0.1406 | 0.5087±0.0401 | 0.2762±0.0175 | 0.3793±0.1897 | 0.5035±0.066 | 0.6563±0.1097 | 0.6134±0.0754 | 0.93±0.0556 | 0.8848±0.031 | 0.7891±0.1866 | 0.8103±0.0445 | 0.3201±0.0819 |
| 30 | 103-45-7 | Acetic acid, 2-phenylethyl ester | 0.1327±0.025 | 0.1458±0.01 | 0.1744±0.0008 | 0.1225±0.0134 | 0.0853±0.0092 | 0.4364±0.472 | 0.1402±0.03 | 0.1854±0.0233 | 0.1751±0.0193 | 0.195±0.014 | 0.1648±0.0149 | 0.2051±0.0465 | 0.273±0.0115 | 0.1076±0.0254 |
| 31 | 23726-93-4 | β-Damascenone | 0.226±0.0389 | 0.2313±0.0177 | 0.2251±0.009 | 0.2407±0.0249 | 0.0562±0.0069 | 0.2251±0.0053 | 0.2331±0.0471 | 0.3001±0.0474 | 0.2999±0.0395 | 0.3566±0.022 | 0.3096±0.0074 | 0.2587±0.0625 | 0.3715±0.0234 | 0.159±0.0393 |
| 32 | 350-03-8 | 3-Acetylpyridine | 0.1182±0.0172 | 0.2445±0.022 | 0.2945±0.0521 | 0.128±0.0116 | 0.0398±0.002 | 0.1432±0.02 | 0.1427±0.039 | 0.1576±0.0114 | 0.1588±0.0291 | 0.2417±0.0087 | 0.2262±0.0076 | 0.2181±0.0789 | 0.2343±0.0223 | 0.1277±0.0196 |
| 33 | 23950-04-1 | α-Nicotine | 0.411±0.069 | 0.5285±0.1507 | 0.6392±0.0987 | 0.388±0.2107 | 0.228±0.052 | 0.4445±0.1804 | 0.6338±0.1459 | 0.3746±0.1083 | 0.5507±0.1016 | 0.5318±0.0609 | 0.8394±0.229 | 0.491±0.2077 | 0.4216±0.082 | 0.1658±0.1002 |
| 34 | 54-11-5 | Nicotine | 353.4507±64.2665 | 483.0067±44.9708 | 376.1275±24.9346 | 347.9182±17.6281 | 144.82±10.8675 | 491.1671±35.3706 | 417.848±55.0332 | 350.8575±67.7698 | 468.0584±61.0831 | 462.7432±23.3558 | 462.1883±16.3259 | 317.3846±36.7011 | 373.2166±33.2941 | 110.5699±27.2023 |
| 35 | 90-05-1 | Phenol, 2-methoxy- | 0.0248±0.0103 | 0.1058±0.0065 | 0.1264±0.0072 | 0.1079±0.0039 | 0.0496±0.003 | 0.107±0.0063 | 0.0206±0.0039 | 0.0223±0.0078 | 0.0312±0.0102 | 0.1291±0.0052 | 0.1096±0.0019 | 0.2634±0.054 | 0.1575±0.0114 | 0.0907±0.0159 |
| 36 | 100-51-6 | Benzyl alcohol | 14.5812±3.3133 | 18.4257±1.407 | 28.0895±0.5195 | 19.6912±0.335 | 14.0909±1.1314 | 20.9973±1.5414 | 20.857±5.7208 | 21.2158±2.0599 | 18.9914±1.5268 | 24.6874±1.2998 | 21.1734±0.6493 | 30.5203±6.6811 | 25.2714±1.8503 | 17.3669±2.225 |
| 37 | 60-12-8 | Phenethyl alcohol | 5.9907±1.2824 | 6.9186±0.4765 | 8.7596±0.0783 | 6.9938±0.3489 | 3.787±0.3759 | 7.1583±0.2811 | 6.7523±1.7139 | 7.775±0.7495 | 7.8356±0.6492 | 8.9248±0.6001 | 7.6002±0.1534 | 11.4842±2.4057 | 11.555±0.6363 | 5.6594±0.9211 |
| 38 | 504-96-1 | Neophytadiene | 114.1211±28.2583 | 132.1617±13.0869 | 116.1177±14.1768 | 107.7492±13.1041 | 44.2906±3.2634 | 102.6759±23.1204 | 100.0785±19.8872 | 145.0829±30.445 | 147.8385±14.1834 | 147.6239±9.7617 | 129.3482±18.1402 | 87.9665±13.9668 | 117.1643±6.4498 | 43.1484±10.5817 |
| 39 | 102608-53-7 | 3,7,11,15-Tetramethyl-2-hexadecen-1-ol | 0.2755±0.0575 | 0.3399±0.0325 | 0.2728±0.0128 | 0.2901±0.0258 | 0.1938±0.0106 | 0.5682±0.0778 | 0.2543±0.0463 | 0.2637±0.0416 | 0.3611±0.0316 | 0.3519±0.0177 | 0.3175±0.0289 | 0.2552±0.0369 | 0.3182±0.0174 | 0.1125±0.0302 |
| **Table A.1** (continued) | | | | | | | | | | | | | | | | |
| 40 | 1072-83-9 | 2-Acetyl pyrrole | 1.1432±0.2444 | 1.4363±0.1047 | 0.9446±0.0042 | 1.113±0.1314 | 0.3716±0.038 | 0.8741±0.066 | 1.0363±0.184 | 1.0064±0.1139 | 1.4985±0.1659 | 1.4763±0.0594 | 1.4212±0.0752 | 2.868±0.5416 | 2.6928±0.2172 | 0.7236±0.2028 |
| 41 | 23267-57-4 | 4-(2,2,6-trimethyl-7-oxabicyclo[4.1.0]hept-1-yl)-3-buten-2-one | 0.6302±0.1243 | 0.8282±0.0596 | 0.6712±0.0153 | 0.7653±0.0993 | 0.4463±0.0275 | 1.0988±0.1314 | 0.613±0.1035 | 0.6394±0.1008 | 0.8316±0.1084 | 0.7775±0.0283 | 0.7586±0.0404 | 0.5749±0.0877 | 0.6405±0.0326 | 0.3305±0.0881 |
| 42 | 108-95-2 | Phenol | 0.1853±0.0387 | 0.3119±0.0286 | 0.2733±0.0025 | 0.4381±0.0515 | 0.1343±0.0203 | 0.3108±0.0238 | 0.2739±0.0518 | 0.2514±0.0319 | 0.2428±0.0223 | 0.4957±0.0354 | 0.3464±0.0344 | 1.823±0.3402 | 0.5423±0.0518 | 0.2386±0.0391 |
| 43 | 2381-87-5 | Dehydromevalonolactone | 0.0465±0.0134 | 0.0468±0.0058 | 0.0547±0.0063 | 0.0385±0.0024 | 0.0221±0.0022 | 0.0629±0.0098 | 0.0423±0.0123 | 0.0442±0.0043 | 0.0594±0.0047 | 0.0678±0.007 | 0.0546±0.0108 | 0.0416±0.0075 | 0.1074±0.0081 | 0.0238±0.0029 |
| 44 | 79-50-5 | DL-Pantolactone | 0.2905±0.051 | 0.3072±0.0234 | 0.2673±0.013 | 0.2608±0.0055 | 0.1307±0.0108 | 0.2387±0.007 | 0.2378±0.0456 | 0.3065±0.0308 | 0.3848±0.0477 | 0.3667±0.0313 | 0.3772±0.0109 | 0.2598±0.048 | 0.6361±0.0418 | 0.1625±0.0231 |
| 45 | 106-44-5 | p-Cresol | 0.1302±0.0261 | 0.2004±0.0164 | 0.3209±0.0077 | 0.2024±0.0213 | 0.0733±0.0041 | 0.2176±0.0096 | 0.218±0.0613 | 0.2251±0.0274 | 0.1712±0.018 | 0.4147±0.0264 | 0.254±0.0117 | 0.732±0.1333 | 0.5461±0.0485 | 0.1518±0.0405 |
| 46 | 502-69-2 | Hexahydrofarnesyl acetone | 0.1819±0.0352 | 0.1542±0.0131 | 0.2755±0.0504 | 0.1242±0.0173 | 0.0299±0.0032 | 0.2095±0.1056 | 0.1657±0.0348 | 0.4554±0.395 | 0.2396±0.025 | 0.379±0.059 | 0.3109±0.0384 | 0.1367±0.0295 | 0.2046±0.0104 | 0.0695±0.0156 |
| 47 | 38818-55-2 | Megastigmatrienone#1 | 0.4705±0.0835 | 0.6186±0.0655 | 0.6374±0.0102 | 0.5613±0.0864 | 0.1648±0.0179 | 0.4858±0.041 | 0.4684±0.0647 | 0.5395±0.0888 | 0.624±0.0843 | 0.845±0.0416 | 0.7124±0.024 | 0.4924±0.0651 | 0.7665±0.0256 | 0.4035±0.0794 |
| 48 | 70901-63-2 | β-Springene | 0.2986±0.0685 | 0.2883±0.0453 | 0.2067±0.0272 | 0.201±0.0204 | 0.0693±0.0067 | 0.2265±0.0484 | 0.1592±0.0406 | 0.2587±0.061 | 0.3886±0.0255 | 0.2562±0.0286 | 0.2359±0.0252 | 0.0899±0.0239 | 0.2173±0.0244 | 0.0798±0.0192 |
| 49 | 532-12-7 | Myosmine | 0.3815±0.0623 | 0.7126±0.0914 | 0.4593±0.0236 | 0.6477±0.187 | 0.3022±0.0504 | 0.4281±0.0304 | 0.4315±0.0743 | 0.3415±0.048 | 0.5078±0.0718 | 0.6439±0.0574 | 0.6253±0.0414 | 0.5772±0.0647 | 0.5175±0.0378 | 0.162±0.0294 |
| 50 | 38818-55-2 | Megastigmatrienone#2 | 1.639±0.3507 | 1.741±0.1482 | 1.35±0.0327 | 1.0213±0.1229 | 0.4407±0.0436 | 1.5037±0.2165 | 1.5025±0.2464 | 1.5417±0.3163 | 2.15±0.2535 | 2.3665±0.307 | 2.0904±0.0333 | 1.8914±0.2584 | 3.2632±0.1397 | 0.7363±0.1862 |
| 51 | 7786-61-0 | 4-Hydroxy-3-methoxystyrene | 0.1155±0.0173 | 0.153±0.0199 | 0.0776±0.0069 | 0.1427±0.0284 | 0.1529±0.1863 | 0.3802±0.0559 | 0.0935±0.0095 | 0.0918±0.0122 | 0.155±0.0267 | 0.0926±0.0116 | 0.0941±0.0288 | 0.1225±0.0051 | 0.0797±0.0084 | 0.079±0.0135 |
| 52 | 54814-64-1 | 2H-Pyran-2-one, 5,6-dihydro-6-pentyl- | 1.2511±0.2071 | 1.1527±0.1819 | 1.53±0.064 | 1.2513±0.183 | 0.2242±0.1373 | 0.7724±0.0284 | 0.9862±0.1727 | 1.043±0.1301 | 1.664±0.2296 | 1.9472±0.0844 | 1.7963±0.0496 | 0.9514±0.1202 | 2.442±0.0472 | 0.3584±0.0843 |
| 53 | 22422-34-0 | (1R,2R,3S,5R)-(-)-2,3-Pinanediol | 0.0421±0.0055 | 0.0117±0.0031 | 0.0634±0.0028 | 0.0559±0.0078 | 0.0215±0.005 | 0.0677±0.0016 | 0.0514±0.009 | 0.1004±0.011 | 0.0567±0.0101 | 0.1117±0.0101 | 0.0856±0.0057 | 0.0225±0.0048 | 0.1342±0.0092 | 0.0347±0.0092 |
| 54 | 112-39-0 | Methyl palmitate | 0.1902±0.0434 | 0.187±0.0104 | 0.1937±0.0121 | 0.1053±0.0024 | 0.0306±0.0057 | 0.0736±0.0086 | 0.2019±0.0262 | 0.1903±0.0305 | 0.2481±0.0251 | 0.2475±0.0011 | 0.251±0.0387 | 0.1713±0.0317 | 0.2259±0.016 | 0.084±0.0187 |
| 55 | 91416-23-8 | Nootkatone | 0.3375±0.0506 | 0.5962±0.1025 | 0.3502±0.0115 | 0.246±0.0294 | 0.0896±0.0129 | 0.2629±0.0399 | 0.311±0.0676 | 0.2214±0.0411 | 0.4399±0.0559 | 0.6013±0.0251 | 0.4313±0.0309 | 0.3667±0.0333 | 0.2775±0.0236 | 0.2228±0.0524 |
| 56 | 38818-55-2 | Megastigmatrienone#3 | 0.245±0.0384 | 0.317±0.0262 | 0.2628±0.0021 | 0.211±0.0455 | 0.0668±0.01 | 0.136±0.0204 | 0.2133±0.0281 | 0.2509±0.0381 | 0.3275±0.0523 | 0.3554±0.0204 | 0.3413±0.0158 | 0.2876±0.0405 | 0.4899±0.024 | 0.1671±0.0323 |
| 57 | 28564-83-2 | 4H-Pyran-4-one, 2,3-dihydro-3,5-dihydroxy-6-methyl- | 0.3901±0.1042 | 0.4149±0.0236 | 0.0908±0.0121 | 0.2694±0.0457 | 0.1922±0.0323 | 0.3888±0.2424 | 0.3192±0.1181 | 0.3464±0.0311 | 0.5037±0.0285 | 0.4473±0.0459 | 0.4019±0.0364 | 0.7988±0.1328 | 0.6034±0.1196 | 0.2935±0.0455 |
| 58 | 20189-42-8 | 3-ethyl-4-methyl-pyrrole-2,5-dione | 0.2556±0.052 | 0.3809±0.0303 | 0.2943±0.0033 | 0.2831±0.0391 | 0.1176±0.0101 | 0.3017±0.0144 | 0.2772±0.0505 | 0.2774±0.0402 | 0.3357±0.035 | 0.4352±0.034 | 0.3812±0.0114 | 0.3867±0.0588 | 0.4739±0.0306 | 0.1898±0.0446 |
| **Table A.1** (continued) | | | | | | | | | | | | | | | | |
| 59 | 38818-55-2 | Megastigmatrienone#4 | 1.1257±0.217 | 1.2026±0.0983 | 1.0318±0.0192 | 0.867±0.0931 | 0.4106±0.0457 | 1.1828±0.1952 | 0.9894±0.1848 | 1.0792±0.1814 | 1.4837±0.1669 | 1.6654±0.1158 | 1.4345±0.0264 | 1.3776±0.2136 | 2.3732±0.1337 | 0.5097±0.1318 |
| 60 | 487-19-4 | Nicotyrine | 10.9544±2.0007 | 21.8467±1.7168 | 12.1554±0.7372 | 15.5619±1.8732 | 3.7458±0.3635 | 13.5055±0.4916 | 12.0349±1.6065 | 11.5031±2.5119 | 14.5149±1.9667 | 20.5254±1.7449 | 16.6218±0.5986 | 15.75±1.2452 | 17.0503±1.6506 | 5.5486±1.4665 |
| 61 | 17092-92-1 | Dihydroactinidiolide | 0.7006±0.1145 | 0.9613±0.1192 | 0.8044±0.0251 | 0.7968±0.1005 | 0.2387±0.0269 | 0.5838±0.047 | 0.7019±0.1024 | 0.768±0.1308 | 0.937±0.1564 | 1.1007±0.0483 | 1.0018±0.0378 | 0.741±0.0977 | 1.0377±0.0491 | 0.5805±0.1309 |
| 62 | 1117-52-8 | (5E,9E)-6,10,14-Trimethylpentadeca-5,9,13-trien-2-one | 0.1332±0.0282 | 0.1168±0.0261 | 0.0728±0.0027 | 0.3079±0.2855 | 0.0213±0.0048 | 0.1603±0.1497 | 0.0937±0.0098 | 0.3265±0.3051 | 0.1746±0.0189 | 0.1599±0.0131 | 0.1622±0.0151 | 0.1027±0.011 | 0.1623±0.0079 | 0.0483±0.0128 |
| 63 | 581-50-0 | 2,3'-Dipyridyl | 0.021±0.0007 | 0.2385±0.0371 | 0.2248±0.0134 | 0.1594±0.0147 | 0.0654±0.0051 | 0.1666±0.0091 | 0.1986±0.041 | 0.1664±0.0273 | 0.0296±0.0095 | 0.0529±0.0062 | 0.2607±0.0208 | 0.1848±0.0218 | 0.2462±0.0064 | 0.0706±0.0135 |
| 64 | 54878-25-0 | Solavetivone | 0.0816±0.0254 | 0.1174±0.0156 | 0.1322±0.0941 | 0.0888±0.0189 | 0.0296±0.0032 | 0.1201±0.0128 | 0.1277±0.0377 | 0.0928±0.0174 | 0.1069±0.0271 | 0.2843±0.0228 | 0.1335±0.0323 | 0.2418±0.2115 | 0.1003±0.0155 | 0.0844±0.0149 |
| 65 | 102488-09-5 | 3-Hydroxy-β-damascone | 0.2593±0.0666 | 0.1767±0.013 | 0.2223±0.0132 | 0.427±0.1225 | 0.1025±0.0091 | 0.361±0.0268 | 0.2271±0.0387 | 0.32±0.0562 | 0.3371±0.0495 | 0.3618±0.0407 | 0.3217±0.0338 | 0.2552±0.0186 | 0.3322±0.0263 | 0.1085±0.034 |
| 66 | 34318-21-3 | 3-oxo-α-ionol,4-(3-hydroxy-1-butenyl)-3,5,5-trimethyl-2-Cyclohexen-1-one | 0.2791±0.1167 | 0.3386±0.0461 | 0.5074±0.0684 | 0.362±0.0811 | 0.1728±0.0473 | 0.4967±0.1052 | 0.5417±0.2363 | 0.6867±0.2212 | 0.3402±0.0622 | 1.0912±0.2141 | 0.4486±0.0512 | 0.4647±0.0171 | 1.2644±0.1443 | 0.2008±0.094 |
| 67 | 57878-30-5 | 5-Hydroxy-3-methyl-2,3-dihydro-1H-inden-1-one | 0.2794±0.0694 | 0.2546±0.0075 | 0.2637±0.0364 | 0.2624±0.043 | 0.0816±0.006 | 0.2163±0.0228 | 0.2633±0.0609 | 0.264±0.0428 | 0.3633±0.0475 | 0.3531±0.0304 | 0.3794±0.026 | 0.251±0.0332 | 0.291±0.028 | 0.1746±0.0469 |

| **Table SA.2** content of volatile compounds of C grade (μg/g) | | | | | | | | | | | | | | | | |
| --- | --- | --- | --- | --- | --- | --- | --- | --- | --- | --- | --- | --- | --- | --- | --- | --- |
| NO. | CAS | Compound | ASXX | BJQXG | GYKY | QNWA | TRST | TRYH | ZYBZ | ZYFG | ZYMT | ZYYQAX | ZYYQSY | HBNS | HNCZ | YNDL |
| 1 | 103-09-3 | 2-Ethylhexyl acetate | 1.1028±0.1543 | 1.2639±0.18 | 0.9895±0.1243 | 1.2768±0.1242 | 0.8187±0.1653 | 0.4435±0.2673 | 1.0135±0.1577 | 0.8334±0.1753 | 1.2982±0.2843 | 1.493±0.2894 | 1.3448±0.2982 | 1.1063±0.0626 | 1.5734±0.1418 | 0.9849±0.1811 |
| 2 | 124-19-6 | 1-Nonanal | 0.1265±0.0666 | 0.0981±0.0447 | 0.0881±0.0178 | 0.0778±0.0055 | 0.0812±0.0258 | 0.0817±0.0053 | 0.1107±0.0444 | 0.1126±0.0228 | 0.1918±0.0432 | 0.0588±0.0091 | 0.0878±0.0079 | 0.1016±0.0053 | 0.0785±0.009 | 0.174±0.0275 |
| 3 | 64-19-7 | Acetic acid | 9.5293±0.8285 | 10.317±0.1292 | 12.5849±1.0761 | 10.6917±0.6257 | 14.6476±3.5784 | 9.4796±0.7822 | 9.5984±1.1395 | 14.023±2.5715 | 14.5081±4.3537 | 15.5365±1.5552 | 12.205±0.7418 | 10.1131±2.1951 | 18.193±0.1911 | 9.4049±0.7964 |
| 4 | 98-01-1 | Furfural | 0.251±0.0121 | 0.1484±0.0162 | 0.1333±0.0364 | 0.1541±0.0298 | 0.1234±0.0555 | 0.1383±0.0168 | 0.2093±0.0081 | 0.1955±0.0442 | 0.1735±0.0373 | 0.1608±0.0266 | 0.1763±0.0301 | 0.1642±0.0125 | 0.2209±0.0218 | 0.4282±0.0268 |
| 5 | 1124-11-4 | Tetramethylpyrazine | 0.1124±0.0163 | 0.0746±0.0132 | 0.0958±0.0356 | 0.1104±0.0153 | 0.0785±0.0397 | 0.0966±0.0245 | 0.0628±0.0081 | 0.0512±0.0175 | 0.0582±0.003 | 0.0952±0.0162 | 0.0684±0.012 | 0.0781±0.0018 | 0.0826±0.0112 | 0.0979±0.0057 |
| 6 | 104-76-7 | 2-Ethylhexanol | 2.3139±0.1389 | 2.2048±0.1334 | 2.3953±0.4234 | 2.5398±0.2912 | 1.4514±0.2461 | 2.0078±0.17 | 2.1038±0.3736 | 1.7028±0.0771 | 2.3604±0.0258 | 2.7983±0.2715 | 2.175±0.233 | 1.7929±0.1176 | 2.3545±0.2214 | 2.0144±0.1121 |
| 7 | 4313-03-5 | 2,4-Heptadienal, (E,E)- | 0.1855±0.0973 | 0.1906±0.0236 | 0.4794±0.1864 | 0.6896±0.1327 | 0.1045±0.0607 | 0.1124±0.0201 | 0.6833±0.0824 | 0.3636±0.0686 | 0.6617±0.199 | 0.6614±0.0775 | 0.57±0.0493 | 0.2636±0.0252 | 0.4192±0.0417 | 0.8241±0.0437 |
| 8 | 100-52-7 | Benzaldehyde | 1.5515±0.7206 | 1.2493±0.0867 | 1.4797±0.4753 | 1.6084±0.21 | 0.8068±0.4018 | 1.0346±0.2248 | 2.8372±0.1591 | 0.6358±0.0624 | 1.455±0.4267 | 1.4483±0.1055 | 0.9745±0.0552 | 0.5688±0.1038 | 0.931±0.025 | 1.1047±0.0985 |
| 9 | 79-09-4 | Propionic acid | 0.1124±0.0203 | 0.1144±0.007 | 0.1219±0.0106 | 0.1158±0.0201 | 0.1919±0.0642 | 0.131±0.0277 | 0.098±0.0159 | 0.1535±0.0485 | 0.1568±0.0664 | 0.1323±0.0399 | 0.1224±0.0086 | 0.1379±0.0292 | 0.2215±0.008 | 0.1348±0.0131 |
| 10 | 78-70-6 | Linalool | 0.3831±0.0209 | 0.36±0.0137 | 0.4292±0.0897 | 0.3956±0.0278 | 0.2772±0.0522 | 0.3361±0.0255 | 0.358±0.0419 | 0.2697±0.012 | 0.3906±0.0325 | 0.3764±0.0703 | 0.3837±0.0394 | 0.3416±0.0069 | 0.4507±0.0208 | 0.5063±0.0262 |
| 11 | 79-31-2 | Isobutyric acid | 0.1993±0.0202 | 0.2111±0.0267 | 0.2068±0.0405 | 0.2419±0.0281 | 0.1451±0.0268 | 0.1425±0.0097 | 0.1938±0.0236 | 0.1465±0.0085 | 0.2108±0.0295 | 0.2569±0.0321 | 0.184±0.0163 | 0.1701±0.003 | 0.2347±0.0213 | 0.1932±0.011 |
| 12 | 1604-28-0 | 6-Meyhyl-3,5-heptadien-2-one | 0.1516±0.035 | 0.1104±0.0166 | 0.1357±0.0527 | 0.2035±0.0585 | 0.0833±0.0331 | 0.0993±0.0145 | 0.1374±0.018 | 0.0791±0.0127 | 0.1238±0.0317 | 0.1576±0.0248 | 0.1407±0.0097 | 0.0842±0.0059 | 0.1424±0.0039 | 0.1224±0.0122 |
| 13 | 432-25-7 | β-Cyclocitral | 0.4727±0.0887 | 0.4336±0.0157 | 0.4023±0.1223 | 0.5064±0.0503 | 0.2856±0.0831 | 0.3356±0.0077 | 0.3717±0.0224 | 0.2642±0.029 | 0.4041±0.076 | 0.5154±0.0803 | 0.4452±0.0368 | 0.3121±0.012 | 0.4933±0.0419 | 0.392±0.0266 |
| 14 | 93-58-3 | Methyl benzoate | 0.4677±0.1324 | 0.3316±0.0073 | 0.4001±0.1668 | 0.5195±0.086 | 0.1799±0.0892 | 0.2697±0.007 | 0.3006±0.034 | 0.1766±0.0243 | 0.2924±0.0861 | 0.4116±0.0364 | 0.3753±0.0113 | 0.1811±0.0035 | 0.3301±0.0336 | 0.1731±0.0227 |
| 15 | 96-48-0 | Butyrolactone | 0.4317±0.0321 | 0.5148±0.009 | 0.5405±0.055 | 0.4781±0.0016 | 0.6736±0.1319 | 0.3204±0.1335 | 0.3328±0.0385 | 0.4326±0.0913 | 0.4646±0.1276 | 0.4579±0.0516 | 0.3679±0.0296 | 0.5076±0.0909 | 0.6339±0.022 | 0.3717±0.0146 |
| 16 | 15356-70-4 | Menthol | 15.0201±2.2861 | 11.7452±0.6151 | 13.8956±3.5564 | 14.597±1.2604 | 12.9694±3.1746 | 12.2636±1.5347 | 12.8067±1.2745 | 8.8328±0.916 | 13.2947±2.2363 | 16.0126±1.7899 | 13.8399±0.945 | 7.6567±0.1946 | 15.3092±0.4832 | 10.6934±0.7086 |
| 17 | 116-26-7 | 1,3-Cyclohexadiene-1-carboxaldehyde, 2,6,6-trimethyl- | 0.1898±0.04 | 0.3131±0.0286 | 0.2956±0.0942 | 0.3764±0.0274 | 0.1668±0.0406 | 0.5772±0.1151 | 0.046±0.0126 | 0.0362±0.0039 | 0.0343±0.0055 | 0.3868±0.0592 | 0.3343±0.0193 | 1.0219±0.3295 | 0.4543±0.0164 | 0.2621±0.0228 |
| 18 | 98-86-2 | Acetophenone | 0.1563±0.0636 | 0.0721±0.0117 | 0.1575±0.0796 | 0.1393±0.0783 | 0.0512±0.018 | 0.0526±0.0124 | 0.1067±0.0485 | 0.0382±0.0094 | 0.0553±0.0225 | 0.0629±0.017 | 0.0735±0.0165 | 0.0274±0.0047 | 0.064±0.0032 | 0.0464±0.0073 |
| 19 | 98-00-0 | 2-Furanmethanol | 0.2838±0.0601 | 0.2775±0.0097 | 0.2232±0.1588 | 0.2773±0.0122 | 0.3846±0.0613 | 0.2919±0.0331 | 0.3033±0.0664 | 0.3373±0.0643 | 0.0521±0.0037 | 0.0454±0.0099 | 0.3442±0.0401 | 0.5983±0.1358 | 0.6265±0.0302 | 0.2392±0.0051 |
| 20 | 116-53-0 | 2-Methyl butyric acid | 0.7599±0.0323 | 0.7322±0.052 | 0.7758±0.1263 | 0.5394±0.0266 | 0.5714±0.0447 | 0.482±0.0187 | 0.5464±0.0612 | 0.5238±0.0225 | 0.2954±0.0692 | 0.3098±0.037 | 0.7079±0.0581 | 0.5383±0.0721 | 0.9006±0.0432 | 0.8212±0.0095 |
| **Table A.2**(continued) | | | | | | | | | | | | | | | | |
| 21 | 1125-21-9 | 2,6,6-Trimethyl-2-cyclohexene-1,4-dione | 0.3587±0.0537 | 0.3333±0.0135 | 0.3451±0.0954 | 0.3702±0.0249 | 0.238±0.0621 | 0.2396±0.0317 | 0.3107±0.0379 | 0.2052±0.0188 | 0.8601±0.0529 | 0.9815±0.165 | 0.3597±0.0181 | 0.2269±0.0031 | 0.4324±0.0198 | 0.3974±0.023 |
| 22 | 695-06-7 | γ-Caprolactone | 0.1331±0.0279 | 0.1359±0.0191 | 0.1397±0.0419 | 0.1462±0.0155 | 0.1905±0.121 | 0.1055±0.0127 | 0.1244±0.0149 | 0.0676±0.0077 | 0.345±0.0724 | 0.4088±0.0453 | 0.0725±0.0093 | 0.0411±0.0095 | 0.1047±0.0051 | 0.0522±0.0034 |
| 23 | 3857-25-8 | 2-Furanmethanol, 5-methyl- | 0.1564±0.0143 | 0.0688±0.0101 | 0.1342±0.0248 | 0.1141±0.008 | 0.159±0.0185 | 0.1274±0.0192 | 0.1324±0.0074 | 0.1362±0.0155 | 0.2134±0.0366 | 0.1655±0.0233 | 0.1622±0.0183 | 0.1937±0.0278 | 0.2222±0.0107 | 0.0993±0.0047 |
| 24 | 54868-48-3 | 6,8-Nonadien-2-one, 8-methyl-5-(1-methylethyl)-, (E)- | 4.277±1.1396 | 4.0973±0.1781 | 4.037±1.4275 | 4.7233±0.5247 | 2.6601±1.0516 | 2.63±0.4749 | 3.8076±0.456 | 2.4933±0.3757 | 3.586±1.0598 | 5.0039±0.5937 | 4.1428±0.3281 | 1.9267±0.1098 | 4.8767±0.1949 | 3.94±0.3497 |
| 25 | 109-52-4 | Valeric acid | 0.1998±0.0226 | 0.1417±0.003 | 0.2374±0.0241 | 0.2608±0.0176 | 0.2452±0.0295 | 0.24±0.0186 | 0.2008±0.0261 | 0.3054±0.0267 | 0.389±0.0728 | 0.2268±0.0158 | 0.238±0.029 | 0.1436±0.0209 | 0.3116±0.0269 | 0.3564±0.0232 |
| 26 | 20547-99-3 | 1,4-Cyclohexanedione, 2,2,6-trimethyl- | 0.0325±0.0055 | 0.0505±0.0051 | 0.0285±0.0075 | 0.034±0.0114 | 0.0284±0.0071 | 0.0219±0.0061 | 0.0247±0.0049 | 0.0168±0.0016 | 0.0308±0.007 | 0.0405±0.0025 | 0.0331±0.0025 | 0.028±0.0004 | 0.0508±0.0019 | 0.0416±0.0041 |
| 27 | 3724-65-0 | Crotonic acid | 0.1341±0.0074 | 0.0986±0.0013 | 0.1934±0.0351 | 0.1309±0.0063 | 0.1954±0.0308 | 0.1055±0.0078 | 0.1487±0.0203 | 0.1449±0.0175 | 0.2±0.0126 | 0.1953±0.0091 | 0.2232±0.0212 | 0.1934±0.0077 | 0.4615±0.0071 | 0.1847±0.0081 |
| 28 | 105-43-1 | 3-Methylvaleric acid | 0.3977±0.0299 | 0.2055±0.0033 | 0.2563±0.0438 | 0.73±0.0157 | 0.1749±0.0159 | 0.1544±0.0014 | 0.3407±0.0299 | 0.264±0.0213 | 0.4956±0.03 | 0.5431±0.0552 | 0.3239±0.028 | 0.2411±0.0083 | 0.5548±0.0118 | 0.3079±0.0147 |
| 29 | 35044-68-9 | 2-Buten-1-one, 1-(2,6,6-trimethyl-1-cyclohexen-1-yl)- | 0.2497±0.2277 | 0.4959±0.0274 | 0.4264±0.1903 | 0.4755±0.0792 | 0.2829±0.1199 | 0.289±0.0451 | 0.3311±0.0261 | 0.2596±0.0482 | 0.3629±0.115 | 0.6636±0.0633 | 0.524±0.049 | 0.2769±0.0111 | 0.6779±0.0255 | 0.3766±0.0247 |
| 30 | 103-45-7 | Acetic acid, 2-phenylethyl ester | 0.1829±0.0479 | 0.2202±0.01 | 0.1216±0.0414 | 0.1095±0.0119 | 0.0754±0.0338 | 0.1177±0.0186 | 0.1114±0.0149 | 0.0804±0.0122 | 0.1083±0.0306 | 0.1645±0.0162 | 0.1198±0.0088 | 0.0822±0.0035 | 0.2509±0.008 | 0.109±0.0093 |
| 31 | 23726-93-4 | β-Damascenone | 0.2463±0.0672 | 0.2636±0.0072 | 0.176±0.0708 | 0.2168±0.0276 | 0.1362±0.0573 | 0.1663±0.0282 | 0.1978±0.0254 | 0.1206±0.0205 | 0.1966±0.0619 | 0.2696±0.0244 | 0.2113±0.0178 | 0.1198±0.0032 | 0.2509±0.008 | 0.1798±0.0164 |
| 32 | 350-03-8 | 3-Acetylpyridine | 0.0779±0.0214 | 0.0827±0.0046 | 0.084±0.0213 | 0.0834±0.0037 | 0.0487±0.0279 | 0.0509±0.0051 | 0.0746±0.0037 | 0.0459±0.0026 | 0.1±0.0419 | 0.1527±0.0255 | 0.1008±0.0035 | 0.0804±0.0073 | 0.1211±0.0194 | 0.1409±0.0042 |
| 33 | 23950-04-1 | α-Nicotine | 0.3428±0.0387 | 0.1429±0.0487 | 0.5603±0.3045 | 0.4064±0.1447 | 0.3699±0.1453 | 0.3998±0.2961 | 0.3338±0.2421 | 0.0667±0.0317 | 0.3565±0.2664 | 0.3225±0.0985 | 0.3971±0.0856 | 0.1322±0.0305 | 0.2824±0.0741 | 0.0901±0.0257 |
| 34 | 54-11-5 | Nicotine | 143.5554±43.7287 | 151.5595±5.0858 | 178.167±61.0139 | 259.6149±32.6369 | 165.5027±48.8888 | 148.328±37.4539 | 143.759±22.9761 | 97.6859±14.9646 | 150.9682±44.5435 | 223.1866±14.596 | 181.5941±12.4491 | 98.2234±7.6737 | 203.409±7.2288 | 65.3022±8.3774 |
| 35 | 90-05-1 | Phenol, 2-methoxy- | 0.0734±0.0127 | 0.0882±0.0055 | 0.0968±0.0283 | 0.0983±0.0097 | 0.0665±0.0184 | 0.0702±0.01 | 0.0234±0.0054 | 0.0191±0.0031 | 0.0196±0.0009 | 0.1027±0.0075 | 0.0951±0.0071 | 0.1511±0.0052 | 0.1839±0.0094 | 0.1289±0.0108 |
| 36 | 100-51-6 | Benzyl alcohol | 17.459±2.4892 | 15.8336±0.5551 | 24.4636±5.0131 | 15.2194±1.0926 | 20.3649±3.3054 | 14.0331±1.8106 | 14.8193±1.3169 | 12.7284±1.071 | 13.6388±1.328 | 18.703±1.2866 | 16.3933±1.0638 | 19.9818±0.0978 | 23.7437±1.0138 | 17.9906±1.1775 |
| 37 | 60-12-8 | Phenethyl alcohol | 5.4539±1.1034 | 5.1177±0.1683 | 7.2554±1.8763 | 5.1162±0.4178 | 5.1907±1.2264 | 4.0695±0.6672 | 4.3905±0.4706 | 6.798±4.9454 | 4.902±0.8258 | 6.9557±0.3596 | 5.5822±0.3968 | 5.5333±0.0896 | 8.6938±0.4151 | 5.9111±0.3704 |
| 38 | 504-96-1 | Neophytadiene | 122.1976±45.305 | 107.4188±16.3032 | 93.8011±36.881 | 140.8233±35.0223 | 72.8573±26.1568 | 62.6986±13.3654 | 71.3306±13.4494 | 53.5665±7.1832 | 86.7197±31.328 | 94.0704±6.5498 | 70.4361±8.8053 | 56.272±5.9474 | 104.6158±10.7042 | 36.6053±3.9329 |
| 39 | 102608-53-7 | 3,7,11,15-Tetramethyl-2-hexadecen-1-ol | 0.2752±0.1065 | 0.2316±0.0337 | 0.2171±0.081 | 0.3335±0.0596 | 0.3316±0.1399 | 0.2308±0.0569 | 0.1639±0.031 | 0.1002±0.0131 | 0.1946±0.0679 | 0.2489±0.01 | 0.1844±0.0165 | 0.1326±0.0164 | 0.2797±0.0214 | 0.1035±0.0111 |
| **Table A.2**(continued) | | | | | | | | | | | | | | | | |
| 40 | 1072-83-9 | 2-Acetyl pyrrole | 1.0223±0.2868 | 1.4172±0.0595 | 0.783±0.275 | 1.0733±0.1262 | 0.8935±0.3016 | 0.6541±0.1268 | 0.9748±0.1061 | 0.7435±0.0947 | 0.9076±0.2384 | 1.6218±0.0976 | 1.2099±0.0981 | 1.4515±0.0879 | 2.9074±0.1309 | 0.6293±0.0347 |
| 41 | 23267-57-4 | 4-(2,2,6-trimethyl-7-oxabicyclo[4.1.0]hept-1-yl)-3-buten-2-one | 0.6631±0.2388 | 0.6342±0.0368 | 0.528±0.2394 | 0.7626±0.1413 | 0.8049±0.3364 | 0.5706±0.145 | 0.4435±0.0785 | 0.2548±0.041 | 0.4891±0.1726 | 0.6234±0.0374 | 0.4612±0.0261 | 0.2534±0.0233 | 0.5903±0.0065 | 0.3046±0.0246 |
| 42 | 108-95-2 | Phenol | 0.2063±0.0602 | 0.5111±0.0236 | 0.2306±0.0704 | 0.4923±0.0594 | 0.2518±0.0891 | 0.282±0.049 | 0.2048±0.0265 | 0.1123±0.013 | 0.2648±0.051 | 0.2858±0.0091 | 0.2618±0.0212 | 1.1174±0.0347 | 0.5742±0.0224 | 0.4266±0.0242 |
| 43 | 2381-87-5 | Dehydromevalonolactone | 0.0266±0.0083 | 0.0167±0.0024 | 0.0241±0.0063 | 0.0248±0.0039 | 0.0059±0.001 | 0.0144±0.0033 | 0.0217±0.0036 | 0.0143±0.0025 | 0.0216±0.0046 | 0.0472±0.0027 | 0.0304±0.0031 | 0.013±0.0018 | 0.0544±0.0007 | 0.0178±0.0022 |
| 44 | 79-50-5 | DL-Pantolactone | 0.187±0.0276 | 0.2301±0.0085 | 0.167±0.0273 | 0.2257±0.0097 | 0.2452±0.0651 | 0.25±0.0351 | 0.1739±0.0198 | 0.1563±0.0125 | 0.2516±0.0217 | 0.1383±0.1714 | 0.2801±0.0349 | 0.1567±0.0065 | 0.5075±0.0359 | 0.1001±0.0033 |
| 45 | 106-44-5 | p-Cresol | 0.1345±0.0482 | 0.2027±0.0069 | 0.1987±0.0673 | 0.1814±0.0225 | 0.1089±0.0425 | 0.1167±0.0288 | 0.139±0.0173 | 0.0701±0.0084 | 0.1204±0.0285 | 0.2512±0.0173 | 0.1883±0.0073 | 0.2927±0.0191 | 0.3458±0.0192 | 0.2341±0.0163 |
| 46 | 502-69-2 | Hexahydrofarnesyl acetone | 0.094±0.0345 | 0.168±0.0816 | 0.1449±0.0554 | 0.1011±0.0384 | 0.039±0.0109 | 0.0473±0.0126 | 0.132±0.0371 | 0.055±0.0044 | 0.1284±0.059 | 0.1509±0.0309 | 0.1688±0.0166 | 0.0371±0.0031 | 0.3458±0.0192 | 0.0481±0.0039 |
| 47 | 38818-55-2 | Megastigmatrienone#1 | 0.448±0.15 | 0.3742±0.0165 | 0.3979±0.1451 | 0.4923±0.0569 | 0.2526±0.0861 | 0.2744±0.0743 | 0.3889±0.0501 | 0.2077±0.0359 | 0.2326±0.1609 | 0.675±0.0412 | 0.3776±0.0378 | 0.1774±0.0201 | 0.5726±0.0115 | 0.3564±0.028 |
| 48 | 70901-63-2 | β-Springene | 0.1829±0.0755 | 0.1095±0.0126 | 0.1479±0.0647 | 0.2361±0.0485 | 0.0653±0.0125 | 0.0793±0.0154 | 0.0983±0.0147 | 0.0571±0.0103 | 0.1275±0.05 | 0.1791±0.008 | 0.1268±0.0159 | 0.0429±0.0041 | 0.1279±0.011 | 0.0736±0.0167 |
| 49 | 532-12-7 | Myosmine | 0.2571±0.0893 | 0.2489±0.019 | 0.2329±0.1038 | 0.2815±0.0422 | 0.3688±0.0053 | 0.175±0.06 | 0.1594±0.0454 | 0.0825±0.0138 | 0.1971±0.061 | 0.3387±0.0261 | 0.2429±0.0178 | 0.1991±0.0149 | 0.2614±0.0224 | 0.1092±0.0043 |
| 50 | 38818-55-2 | Megastigmatrienone#2 | 1.0031±0.3614 | 1.1142±0.0339 | 0.8983±0.3445 | 0.931±0.1455 | 0.6637±0.225 | 0.7287±0.1808 | 0.9553±0.156 | 0.5002±0.0804 | 1.0083±0.3039 | 1.6262±0.0494 | 1.2141±0.1234 | 0.6626±0.0541 | 2.1788±0.0721 | 0.6087±0.0373 |
| 51 | 7786-61-0 | 4-Hydroxy-3-methoxystyrene | 0.1483±0.0439 | 0.1735±0.0133 | 0.1071±0.0429 | 0.1647±0.0211 | 0.0452±0.0158 | 0.0588±0.0108 | 0.1472±0.0224 | 0.0838±0.0121 | 0.1416±0.0411 | 0.1588±0.0061 | 0.1246±0.0215 | 0.0695±0.0097 | 0.1315±0.0136 | 0.0855±0.0035 |
| 52 | 54814-64-1 | 2H-Pyran-2-one, 5,6-dihydro-6-pentyl- | 0.4943±0.1155 | 0.3156±0.0242 | 0.6±0.2451 | 0.6046±0.1063 | 0.4179±0.1325 | 0.3856±0.1021 | 0.3657±0.0873 | 0.2104±0.0295 | 0.4231±0.1469 | 1.0229±0.0652 | 0.8493±0.051 | 0.1354±0.005 | 1.2225±0.0631 | 0.1929±0.02 |
| 53 | 22422-34-0 | (1R,2R,3S,5R)-(-)-2,3-Pinanediol | 0.0321±0.0106 | 0.0253±0.0013 | 0.0504±0.0179 | 0.0738±0.0086 | 0.0686±0.0187 | 0.0683±0.0192 | 0.066±0.0091 | 0.0299±0.0032 | 0.0915±0.0269 | 0.084±0.0054 | 0.0774±0.0062 | 0.0131±0.0006 | 0.1403±0.005 | 0.0933±0.0128 |
| 54 | 112-39-0 | Methyl palmitate | 0.1342±0.0481 | 0.1437±0.0392 | 0.1409±0.0571 | 0.1231±0.0142 | 0.1181±0.0529 | 0.0572±0.0191 | 0.1199±0.0228 | 0.0833±0.0132 | 0.1448±0.0537 | 0.2074±0.0395 | 0.1312±0.0065 | 0.0774±0.0126 | 0.2449±0.0121 | 0.0853±0.0088 |
| 55 | 91416-23-8 | Nootkatone | 0.1537±0.0532 | 0.1567±0.017 | 0.1551±0.0717 | 0.1151±0.0184 | 0.0251±0.0125 | 0.0695±0.017 | 0.1504±0.0166 | 0.051±0.0099 | 0.1115±0.035 | 0.2635±0.0209 | 0.1818±0.012 | 0.0575±0.0026 | 0.1988±0.0163 | 0.1503±0.0063 |
| 56 | 38818-55-2 | Megastigmatrienone#3 | 0.102±0.0362 | 0.156±0.0162 | 0.1718±0.0622 | 0.1785±0.015 | 0.0987±0.0395 | 0.0648±0.0148 | 0.1359±0.0254 | 0.0752±0.0118 | 0.1519±0.0564 | 0.2524±0.0075 | 0.1854±0.0184 | 0.0677±0.006 | 0.2996±0.0211 | 0.1359±0.004 |
| 57 | 28564-83-2 | 4H-Pyran-4-one, 2,3-dihydro-3,5-dihydroxy-6-methyl- | 0.3672±0.1085 | 0.4269±0.0425 | 0.1918±0.0599 | 0.13±0.0778 | 0.3365±0.0559 | 0.2159±0.037 | 0.2732±0.0287 | 0.2094±0.0301 | 0.3262±0.0243 | 0.4766±0.0399 | 0.4412±0.0537 | 0.491±0.037 | 0.7693±0.0741 | 0.0933±0.0155 |
| 58 | 20189-42-8 | 3-ethyl-4-methyl-pyrrole-2,5-dione | 0.2468±0.0792 | 0.2693±0.0076 | 0.1976±0.0745 | 0.2156±0.0247 | 0.1507±0.0511 | 0.1776±0.0422 | 0.1907±0.0259 | 0.1072±0.0163 | 0.1954±0.052 | 0.3351±0.0211 | 0.2351±0.014 | 0.1298±0.0088 | 0.3691±0.0138 | 0.1755±0.0121 |
| 59 | 38818-55-2 | Megastigmatrienone#4 | 0.7616±0.2699 | 0.7661±0.0409 | 0.7084±0.263 | 0.7654±0.1099 | 0.5672±0.2036 | 0.6011±0.1503 | 0.6406±0.0918 | 0.3685±0.0567 | 0.701±0.2281 | 1.222±0.0623 | 0.87±0.0749 | 0.5254±0.0448 | 1.653±0.0418 | 0.4095±0.0255 |
| **Table A.2**(continued) | | | | | | | | | | | | | | | | |
| 60 | 487-19-4 | Nicotyrine | 8.1902±2.6699 | 8.2634±0.3574 | 8.566±3.3737 | 10.079±1.427 | 4.9457±1.7604 | 6.3168±2.2154 | 5.0522±0.8708 | 2.955±0.4444 | 5.6015±1.7436 | 11.3023±0.6664 | 7.8408±0.4397 | 4.5793±0.2479 | 10.2054±0.4534 | 3.5367±0.4586 |
| 61 | 17092-92-1 | Dihydroactinidiolide | 0.7718±0.265 | 1.0388±0.0683 | 0.603±0.2446 | 0.7172±0.1043 | 0.5319±0.1658 | 0.4571±0.1166 | 0.7352±0.1134 | 0.3901±0.0606 | 0.7469±0.2405 | 0.95±0.0522 | 0.7043±0.0531 | 0.3617±0.0363 | 1.0177±0.0155 | 0.5909±0.0273 |
| 62 | 1117-52-8 | (5E,9E)-6,10,14-Trimethylpentadeca-5,9,13-trien-2-one | 0.0514±0.0162 | 0.388±0.4304 | 0.0631±0.0365 | 0.0801±0.021 | 0.0262±0.0112 | 0.0339±0.0134 | 0.0617±0.0127 | 0.0956±0.0815 | 0.0895±0.0418 | 0.1072±0.0084 | 0.0861±0.0137 | 0.0363±0.004 | 0.09±0.0062 | 0.0426±0.0013 |
| 63 | 581-50-0 | 2,3'-Dipyridyl | 0.109±0.0278 | 0.1208±0.0319 | 0.1335±0.0347 | 0.1066±0.0121 | 0.0856±0.0401 | 0.0822±0.014 | 0.0888±0.0152 | 0.0506±0.005 | 0.096±0.0292 | 0.1966±0.0322 | 0.1823±0.0172 | 0.0536±0.0073 | 0.1569±0.0158 | 0.0523±0.0045 |
| 64 | 54878-25-0 | Solavetivone | 0.0676±0.0478 | 0.0696±0.0149 | 0.4157±0.4691 | 0.0523±0.0087 | 0.0421±0.0168 | 0.0263±0.0211 | 0.056±0.0241 | 0.0255±0.003 | 0.0642±0.0399 | 0.112±0.0203 | 0.0948±0.0172 | 0.0301±0.0036 | 0.0871±0.0077 | 0.0654±0.0055 |
| 65 | 102488-09-5 | 3-Hydroxy-β-damascone | 0.3903±0.1392 | 0.1606±0.0084 | 0.2427±0.1012 | 0.3031±0.0462 | 0.1391±0.0528 | 0.2561±0.0546 | 0.4931±0.1872 | 0.1195±0.0205 | 0.2163±0.0746 | 0.3042±0.0516 | 0.2421±0.0206 | 0.1322±0.0133 | 0.313±0.0144 | 0.0942±0.0202 |
| 66 | 34318-21-3 | 3-oxo-α-ionol,4-(3-hydroxy-1-butenyl)-3,5,5-trimethyl-2-Cyclohexen-1-one | 0.2119±0.0587 | 0.2103±0.0347 | 0.2807±0.107 | 0.3712±0.0949 | 0.3228±0.2848 | 0.2815±0.0501 | 0.2124±0.0622 | 0.213±0.0533 | 0.2776±0.1098 | 0.4099±0.2001 | 0.2934±0.1099 | 0.3722±0.0327 | 0.7233±0.0931 | 0.3456±0.1617 |
| 67 | 57878-30-5 | 5-Hydroxy-3-methyl-2,3-dihydro-1H-inden-1-one | 0.1977±0.0636 | 0.2426±0.004 | 0.1896±0.0654 | 0.2335±0.0399 | 0.1397±0.0461 | 0.1428±0.0356 | 0.2264±0.0242 | 0.1013±0.0132 | 0.2373±0.0787 | 0.3538±0.0199 | 0.2635±0.0058 | 0.1215±0.0096 | 0.2477±0.0069 | 0.1637±0.0169 |

| **Table SA.3** OAV of volatile compounds of different origins | | | | | | | | | |
| --- | --- | --- | --- | --- | --- | --- | --- | --- | --- |
|  | 1-Nonanal | Benzaldehyde | β-cyclocitral | γ-Caprolactone | 6,8-Nonadien-2-one, 8-methyl-5-(1-methylethyl)-, (E)- | Phenol, 2-methoxy- | Benzyl alcohol | Phenethyl alcohol | p-Cresol |
| ASXX-B | 26.3 | 2.8 | 24.3 | 0.8 | 2.4 | 1.2 | 5.7 | 10.7 | 13.0 |
| ASXX-C | 42.2 | 2.1 | 24.9 | 0.5 | 2.3 | 3.7 | 6.9 | 9.7 | 13.4 |
| BJQXG-B | 64.6 | 2.4 | 32.3 | 0.6 | 4.1 | 5.3 | 7.3 | 12.4 | 20.0 |
| BJQXG-C | 32.7 | 1.7 | 22.8 | 0.5 | 2.3 | 4.4 | 6.2 | 9.1 | 20.3 |
| GYKY-B | 27.7 | 3.1 | 29.2 | 0.8 | 3.4 | 6.3 | 11.1 | 15.6 | 32.1 |
| GYKY-C | 29.4 | 2.0 | 21.2 | 0.5 | 2.2 | 4.8 | 9.6 | 13.0 | 19.9 |
| QNWA-B | 33.9 | 2.5 | 30.9 | 0.7 | 3.2 | 5.4 | 7.8 | 12.5 | 20.2 |
| QNWA-C | 25.9 | 2.1 | 26.7 | 0.6 | 2.6 | 4.9 | 6.0 | 9.1 | 18.1 |
| TRST-B | 51.6 | 0.8 | 14.8 | 0.3 | 0.9 | 2.5 | 5.5 | 6.8 | 7.3 |
| TRST-C | 27.1 | 1.1 | 15.0 | 0.7 | 1.5 | 3.3 | 8.0 | 9.3 | 10.9 |
| TRYH-B | 34.5 | 2.2 | 28.9 | 0.8 | 2.4 | 5.4 | 8.3 | 12.8 | 21.8 |
| TRYH-C | 27.2 | 1.4 | 17.7 | 0.4 | 1.4 | 3.5 | 5.5 | 7.3 | 11.7 |
| ZYBZ-B | 30.4 | 4.3 | 25.0 | 0.6 | 2.8 | 1.0 | 8.2 | 12.1 | 21.8 |
| ZYBZ-C | 36.9 | 3.8 | 19.6 | 0.5 | 2.1 | 1.2 | 5.8 | 7.8 | 13.9 |
| ZYFG-B | 25.3 | 1.8 | 26.2 | 0.6 | 3.1 | 1.1 | 8.4 | 13.9 | 22.5 |
| ZYFG-C | 37.5 | 0.8 | 13.9 | 0.3 | 1.4 | 1.0 | 5.0 | 12.1 | 7.0 |
| ZYMT-B | 33.7 | 2.7 | 31.6 | 2.2 | 3.1 | 1.6 | 7.5 | 14.0 | 17.1 |
| ZYMT-C | 63.9 | 1.9 | 21.3 | 1.3 | 2.0 | 1.0 | 5.4 | 8.8 | 12.0 |
| ZYYQAX-B | 22.9 | 3.2 | 33.7 | 0.6 | 3.7 | 6.5 | 9.7 | 15.9 | 41.5 |
| ZYYQAX-C | 19.6 | 1.9 | 27.1 | 1.6 | 2.7 | 5.1 | 7.4 | 12.4 | 25.1 |
| ZYYQSY-B | 30.1 | 2.9 | 31.6 | 0.6 | 3.8 | 5.5 | 8.3 | 13.6 | 25.4 |
| ZYYQSY-C | 29.3 | 1.3 | 23.4 | 0.3 | 2.3 | 4.8 | 6.5 | 10.0 | 18.8 |
| HBNS-B | 22.0 | 2.3 | 33.2 | 0.3 | 2.8 | 13.2 | 12.0 | 20.5 | 73.2 |
| HBNS-C | 33.9 | 0.8 | 16.4 | 0.2 | 1.1 | 7.6 | 7.9 | 9.9 | 29.3 |
| HNCZ-B | 26.2 | 1.8 | 30.2 | 0.6 | 3.2 | 7.9 | 9.9 | 20.6 | 54.6 |
| HNCZ-C | 26.2 | 1.2 | 26.0 | 0.4 | 2.7 | 9.2 | 9.3 | 15.5 | 34.6 |
| YNDL-B | 100.4 | 1.2 | 21.0 | 0.2 | 2.4 | 4.5 | 6.8 | 10.1 | 15.2 |
| YNDL-C | 58.0 | 1.5 | 20.6 | 0.2 | 2.2 | 6.4 | 7.1 | 10.6 | 23.4 |

**The code (A.1)**

from sklearn.model_selection import train_test_split

from sklearn.linear_model import LogisticRegression

from sklearn.metrics import (

accuracy_score, recall_score, precision_score, f1_score,

roc_auc_score, confusion_matrix

)

from sklearn.preprocessing import LabelEncoder

import numpy as np

# Encode the 'group' column: YES -> 1, NO -> 0

label_encoder = LabelEncoder()

df['group'] = label_encoder.fit_transform(df['group'])

# Split into features and target

X = df.drop(columns=['group'])

y = df['group']

# Train-test split

X_train, X_test, y_train, y_test = train_test_split(X, y, test_size=0.2, random_state=42)

# Train logistic regression model

model = LogisticRegression(max_iter=1000)

model.fit(X_train, y_train)

# Predictions

y_pred = model.predict(X_test)

y_prob = model.predict_proba(X_test)[:, 1]

# Evaluation metrics

accuracy = accuracy_score(y_test, y_pred)

recall = recall_score(y_test, y_pred)

precision = precision_score(y_test, y_pred)

f1 = f1_score(y_test, y_pred)

auc = roc_auc_score(y_test, y_prob)

# Sensitivity = Recall (already calculated)

# Specificity = TN / (TN + FP)

tn, fp, fn, tp = confusion_matrix(y_test, y_pred).ravel()

specificity = tn / (tn + fp)

# Most informative feature (highest absolute coefficient)

feature_importance = pd.Series(model.coef_[0], index=X.columns)

most_informative = feature_importance.abs().sort_values(ascending=False).head(1)

{

"Accuracy": accuracy,

"Sensitivity (Recall)": recall,

"Specificity": specificity,

"Precision": precision,

"F1 Score": f1,

"AUC": auc,

"Most Informative Feature": most_informative.to_dict()

}

from sklearn.model_selection import cross_val_score, cross_val_predict

from sklearn.metrics import make_scorer

# Define custom scorers

scoring = {

'accuracy': 'accuracy',

'recall': 'recall',

'precision': 'precision',

'f1': 'f1',

'roc_auc': 'roc_auc'

}

# Initialize logistic regression

model = LogisticRegression(max_iter=1000)

# Perform cross-validation

cv_scores = {}

for metric, scorer in scoring.items():

scores = cross_val_score(model, X, y, cv=5, scoring=scorer)

cv_scores[metric] = {

'mean': scores.mean(),

'std': scores.std(),

'all_scores': scores

}

cv_scores
